# The HSP70 co-chaperone DNAJC14 targets misfolded pendrin for unconventional protein secretion

**DOI:** 10.1038/ncomms11386

**Published:** 2016-04-25

**Authors:** Jinsei Jung, Jiyoon Kim, Shin Hye Roh, Ikhyun Jun, Robert D. Sampson, Heon Yung Gee, Jae Young Choi, Min Goo Lee

**Affiliations:** 1 Department of Pharmacology and Brain Korea 21 Project for Medical Sciences, Yonsei University College of Medicine, Seoul 120-752, Republic of Korea; 2 Department of Otorhinolaryngology, Yonsei University College of Medicine, Seoul 120-752, Republic of Korea; 3 Division of Nephrology, Department of Medicine, Boston Children's Hospital and Harvard Medical School, Boston, Massachusetts 02115, USA

## Abstract

Mutations in *SLC26A4*, which encodes pendrin, are responsible for hearing loss with an enlarged vestibular aqueduct and Pendred syndrome. The most prevalent mutation in East Asia is p.H723R (His723Arg), which leads to defects in protein folding and cell-surface expression. Here we show that H723R-pendrin can be rescued to the cell surface by an HSP70 co-chaperone DNAJC14-dependent unconventional trafficking pathway. Blockade of ER-to-Golgi transport or activation of ER stress signals induced Golgi-independent cell-surface expression of H723R-pendrin and restored its cell-surface Cl^−^/HCO_3_^−^ exchange activity. Proteomic and short interfering RNA screenings with subsequent molecular analyses showed that Hsc70 and DNAJC14 are required for the unconventional trafficking of H723R-pendrin. Moreover, DNAJC14 upregulation was able to induce the unconventional cell-surface expression of H723R-pendrin. These results indicate that Hsc70 and DNAJC14 play central roles in ER stress-associated unconventional protein secretion and are potential therapeutic targets for diseases such as Pendred syndrome, which arise from transport defects of misfolded proteins.

## Abstract (web summary)

Mutations in pendrin, a plasma membrane transporter, lead to Pendred syndrome, which is associated with hearing loss. Here, Jung *et al*. show that cell-surface expression of a mutated form of pendrin can be restored by blocking ER-to-Golgi traffic and triggering a DNAJC14 dependent unconventional secretion pathway.

## Graphical abstract

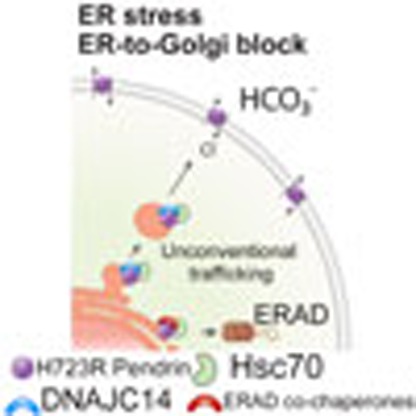

Pendrin encoded by the *SLC26A4* gene is a transmembrane protein that transports monovalent anions, such as Cl^−^, I^−^, HCO_3_^−^, and formate in the inner ear, thyroid follicles and renal cortical collecting ducts^1^. Mutations in *SLC26A4* cause non-syndromic recessive deafness with an enlarged vestibular aqueduct (DFNB4, [MIM 600791]) and Pendred syndrome (PDS, [MIM 274600]), and are considered to be the most common cause of hereditary hearing loss in East Asia and the second most common cause worldwide^2^,^3^,^4^,^5^,^6^,^7^. Specifically, p.H723R (His723Arg) is the most prevalent pathological mutation in DFNB4 and PDS, representing about 60% of presentations in Korea and Japan^8^,^9^. The H723R mutation results in protein misfolding, retention in the endoplasmic reticulum (ER), and finally, degradation by the ER-associated degradation (ERAD) pathway^10^. Consequently, negligible amounts of H723R-pendrin reach the plasma membrane, and most of the Cl^−^/ HCO_3_^−^ or Cl^−^/I^−^ exchange activity at the cell surface is lost.

The folding of secretory and transmembrane proteins is monitored by the ER quality control (ERQC) systems. During ERAD, non-native or misfolded proteins that do not pass ERQC are retrotranslocated to the cytoplasm and degraded by the ubiquitin–proteasome machinery^11^. Molecular chaperones and associated factors play critical roles in the recognition of ERAD target substrates. The heat shock cognate protein 70 (Hsc70; encoded by *HSPA8*), a member of heat shock 70 kDa proteins (HSP70s), is one of the premier chaperones involved in this process. Although Hsc70 is a cytosolic chaperone, because most membrane proteins have large cytosolic domains, Hsc70 binds to and censors the folding status of substrate membrane proteins that are synthesized in the ER and transported to the cell surface via the conventional ER-to-Golgi secretion pathway. For example, Hsc70 has been shown to promote folding of the cystic fibrosis transmembrane conductance regulator (CFTR) Cl^−^ channel and also to be involved in the ERAD of the folding-deficient Phe508-deleted (ΔF508) CFTR, the most common cystic fibrosis-causing mutation worldwide^12^.

The HSP70 chaperones do not function alone. In fact, co-chaperones of Hsc70, such as Hdj-2 and CHIP, have been shown to participate in the folding, biogenesis and degradation of CFTR^13^,^14^. Notably, the HSP70 proteins not only are involved in protein folding and degradation but also function in a myriad of biological processes, including protein–protein interaction and intracellular trafficking of various proteins^15^,^16^. This multitude of roles is not easily reconciled with the simple activity of HSP70s in ATP-dependent substrate protein binding and release cycles. Consequently, it has been suggested that much of the functional diversity of HSP70 is driven by a diverse class of cofactors, such as J proteins, also known as HSP40s (ref. 15).

Recently, it was found that ΔF508-CFTR can be expressed on the cell surface via an unconventional secretion pathway under specific cellular conditions^17^. Blockade of ER-to-Golgi transport induces ER stress and unfolded protein response due to accumulation of excessive nascent, unfolded polypeptides in the ER^18^. Interestingly, ER-to-Golgi blockade or activation of ER stress signals induced the plasma membrane expression of ΔF508-CFTR via a Golgi-bypass trafficking pathway and restored CFTR Cl^−^ channel activity at the cell surface^17^. Although Golgi reassembly and stacking proteins (GRASPs) were identified as a cargo-recruiting factor in this unconventional transport of ΔF508-CFTR, the mechanisms underlying ER-to-Golgi blockade or ER stress-associated unconventional protein secretion pathway are largely unknown.

Here we report that the clinically important, folding-deficient membrane protein H723R-pendrin can be rescued to the cell surface by the unconventional protein secretion pathway. More importantly, the HSP70 co-chaperone DNAJC14 plays an essential role in allowing H723R-pendrin to escape ERAD and be expressed on the cell surface. These results provide insights into the mechanism of ER stress-associated unconventional protein secretion. Furthermore, identification of DNAJC14 as a factor targeting entry of a folding-deficient membrane protein into the unconventional protein secretion pathway will have an impact on the development of therapeutic strategies for diseases arising from transport defects of misfolded proteins.

## Results

### Unconventional secretion of pendrin by ER-to-Golgi blockade

**Figure 1:**
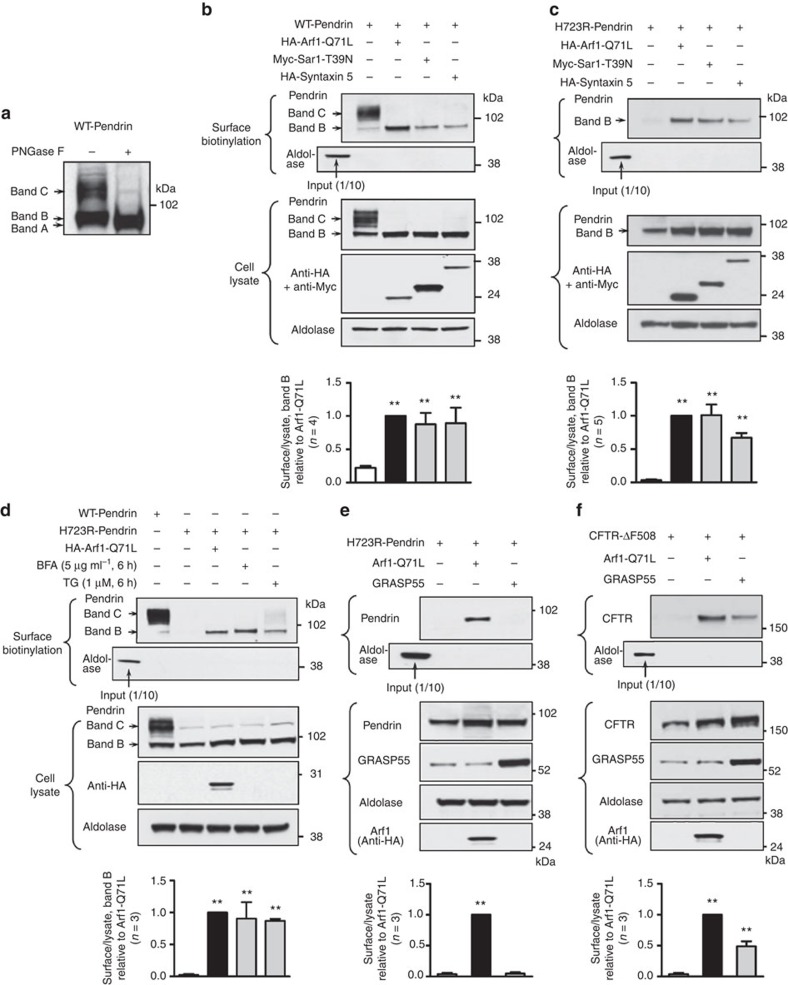
Cell-surface expression of H723R-pendrin by unconventional protein secretion. (**a**) PANC-1 cells were transfected with plasmids encoding wild-type (WT)-pendrin. Protein samples were treated with *N*-glycosidase F (PNGase F), which deglycosylates all *N*-glycan chains and immunoblotted with anti-pendrin antibodies. Band A: deglycosylated pendrin, band B: immature core-glycosylated pendrin, band C: mature complex-glycosylated pendrin. (**b**,**c**) Surface biotinylation assays were performed in PANC-1 cells expressing WT- (**b**) or H723R- (**c**) pendrin. In control cells (first lanes in **b** and **c**), the band C form of WT-pendrin and no form of H723-pendrin were expressed on the cell surface. Blockade of ER-to-Golgi traffic by overexpression of Arf1-Q71L, Sar1-T39N or syntaxin 5 induced the cell-surface expression of core-glycosylated (band B) WT- and H723R-pendrins. The absence of the cytosolic protein aldolase A in the biotinylated fraction confirmed cell surface protein-specific labelling in each experiment (input: 40 μg of cell lysate, surface biotinylation: 400 μg protein). (**d**) Treatments with brefeldin A (BFA) or thapsigargin (TG) also induced cell-surface expression of H723R-pendrin in PANC-1 cells. (**e**,**f**) Whereas GRASP55 overexpression induced cell-surface expression of ΔF508-CFTR in PANC-1 cells (**f**), GRASP55 was not effective in inducing cell-surface expression of H723R-pendrin (**e**). Quantitation of multiple experiments is presented under each immunoblot. ^**^P<0.01 by one-way analysis of variance, compared with lane 1, the number of replicates (*n*) is presented in each panel. Unprocessed original scans of western blots are shown in Supplementary Fig. 10.

Pendrin is a glycosylated membrane protein that undergoes ER core-glycosylation (band B, ∼84 kDa, Fig. 1a) and Golgi complex-glycosylation (band C, ∼120 kDa) during conventional ER-to-Golgi exocytosis, with the non-glycosylated nascent polypeptides (band A, ∼82 kDa) being barely detectable in the immunoblot of cytosolic samples. It is commonly assumed that the mature complex-glycosylated pendrin is the only form expressed in the plasma membrane^10^. However, in the case of CFTR, the immature ER form of core-glycosylated protein can be unconventionally expressed on the cell surface by ER-to-Golgi blockade^17^. Therefore, we examined whether a similar phenomenon exists with pendrin. PANC-1 cells derived from pancreatic ducts were used to examine the cell-surface expression of pendrin. Because these cells retain epithelial properties and do not express detectable levels of endogenous pendrin, they constitute a suitable model for studying the intracellular trafficking of pendrin through transfection of plasmids encoding the mutant protein.

Inhibition of ER-to-Golgi traffic was achieved by expression of the dominant-negative forms of Arf1 (a component of the coat protein complex [COP]I) and Sar1 (a component of COPII), or by overexpression of syntaxin 5 (required for the fusion of COPII transport vesicles with the acceptor Golgi membrane)^17^. As shown in Fig. 1b, when wild-type (WT) pendrin was expressed in PANC-1 cells, the band C form of complex-glycosylated pendrin was mainly expressed on the cell surface. Blockade of ER-to-Golgi transport induced by the Arf1-Q71L and Sar1-T39N dominant-negative mutants or overexpressed syntaxin 5 completely blocked the Golgi maturation of WT-pendrin glycosylation. Interestingly, even when the ER-to-Golgi transport was blocked, the band B form of core-glycosylated WT-pendrin continued to reach the plasma membrane (Fig. 1b). More importantly, the folding-deficient H723R-pendrin, which did not travel to the cell surface in control cells, reached the cell surface when ER-to-Golgi trafficking was blocked (Fig. 1c).

Similar to the case of ΔF508-CFTR^17^, ER stress-inducing agents, such as brefeldin A (BFA) and thapsigargin, also induced the cell-surface expression of core-glycosylated H723R-pendrin (Fig. 1d). Furthermore, depletion of three major ER stress-signal transducers, IRE1α, PERK and ATF6 (ref. 19), by treatment with specific siRNAs revealed that the IRE1α-mediated signalling arm is involved in Arf1-Q71L-induced exocytosis of H723R-pendrin (Supplementary Fig. 1). These results suggest that ER stress-related signals are required for the unconventional secretion of core-glycosylated pendrins. Unlike ΔF508-CFTR, in which the unconventional cell-surface expression is dependent on GRASP^17^, cell-surface expression of H723R-pendrin was neither inhibited by depletion of GRASPs (Supplementary Fig. 2a) nor induced by overexpression of GRASP55 (Fig. 1e; Supplementary Fig. 2b). As a control, GRASP55-mediated induction of cell-surface expression of ΔF508-CFTR was recapitulated in this study using PANC-1 cells (Fig. 1f; Supplementary Fig. 2c).

**Figure 2:**
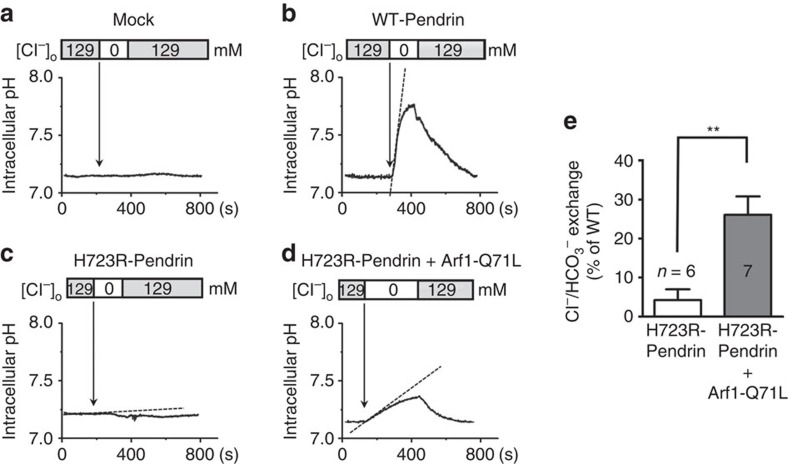
Rescue of anion exchange activity of H723R-pendrin by Arf1-Q71L. The Cl^−^/HCO_3_^−^ exchange activity was measured by recording pH_i_ as detailed in Methods. A clone of HEK 293 cells that exhibited minimal intrinsic anion (Cl^−^/HCO_3_^−^) exchange activity (**a**) was transfected with the indicated plasmids (**b**–**d**) and then anion exchange activity was analysed. The quantitation of multiple experiments is depicted in **e**. Blockade of ER-to-Golgi trafficking by Arf1-Q71L significantly increased Cl^−^/HCO_3_^−^ exchange activity in cells expressing H723R-pendrin. ^**^P<0.01 by unpaired Student's *t*-test, the number of replicates (*n*) is presented in each lane.

To determine whether the core-glycosylated H723R-pendrin transported to the cell surface was functional, the pendrin-dependent Cl^−^/HCO_3_^−^ exchange activity was examined in a clone of HEK 293 cells that has minimal intrinsic Cl^−^/HCO_3_^−^ exchange activity (Fig. 2a–e). The Cl^−^/HCO_ 3_^−^ exchange activity was measured in cells attached to coverslips by recording pH_i_ in response to [Cl^−^]_o_ changes of the perfusate. Strong Cl^−^/HCO_3_^−^ exchange activity was elicited by transfection with WT-pendrin (0.230±0.029 ΔpH unit min^−1^, *n*=13), but not H723R-pendrin (0.010±0.006 ΔpH unit min^−1^, *n*=6). Notably, blockade of the ER-to-Golgi transport by Arf1-Q71L significantly increased the H723R-pendrin-mediated Cl^−^/HCO_3_^−^ exchange activity to 0.060±0.011 ΔpH unit min^−1^ (*n*=7, *P*<0.01), which corresponded to 26% of the WT-pendrin activity. Taken together, these results indicate that ER-to-Golgi blockade or ER stress induces the functional cell-surface expression of core-glycosylated H723R-pendrin via a GRASP-independent route that bypasses the Golgi.

### Hsc70 is required for unconventional secretion of pendrin

**Figure 3:**
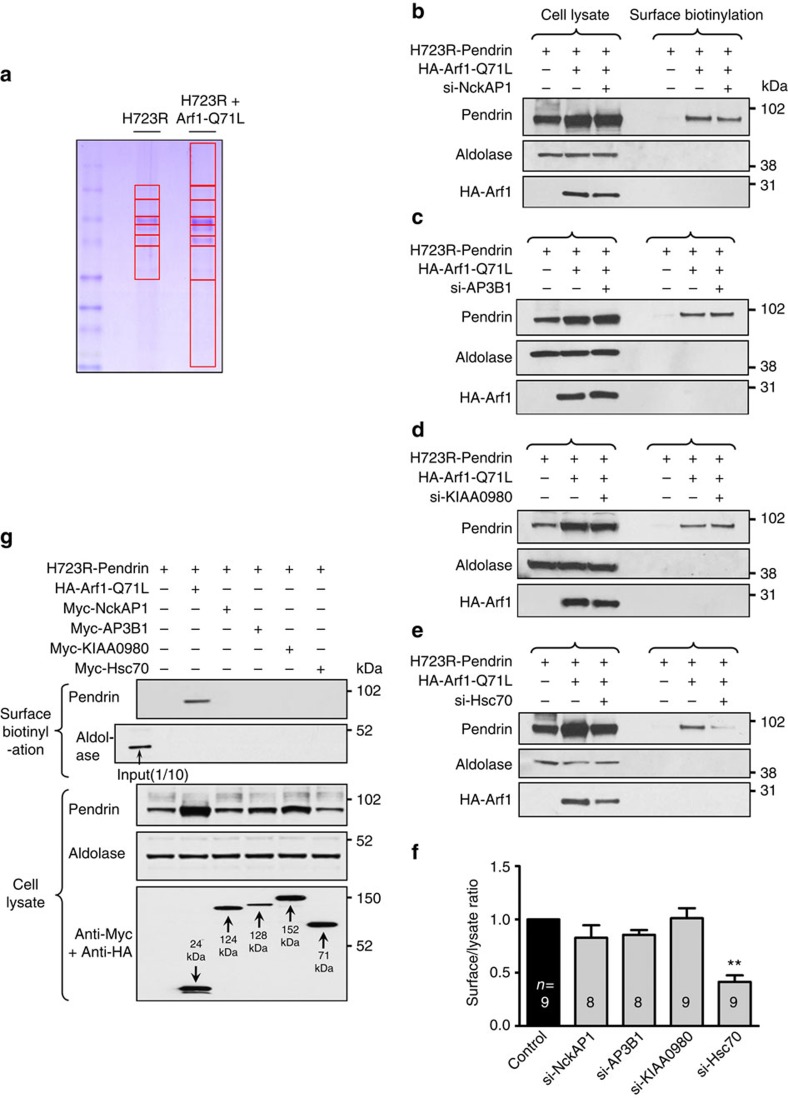
Hsc70 is necessary for Arf1-Q71L–mediated unconventional cell-surface expression of H723R-pendrin. (**a**) After tandem affinity purification, binding partners of H723R-pendrin in PANC-1 cells with or without Arf1-Q71L co-expression were separated by electrophoresis. Subsequent analyses by LC-MS/MS identified NckAP1, AP3B1, KIAA0980 and Hsc70 as binding partners of H723R-pendrin (Supplementary Table 1). (**b**–**f**) Surface biotinylation assays were performed after siRNA-mediated knockdown of indicated candidate proteins. Representative blots are shown in **b**–**e** and a summary of multiple experiments is presented in **f**. Only treatment with siRNA targeting Hsc70 showed a significant effect. ^**^P<0.01 by one-way analysis of variance, difference from control, the number of replicates (*n*) is presented in each lane. (**g**) Surface biotinylation assays were performed after overexpression of NckAP1, AP3B1, KIAA0980 and Hsc70. None of the candidate proteins induced cell-surface expression of H723R-pendrin. The data are representative of three independent experiments. Unprocessed original scans of western blots are shown in Supplementary Fig. 10.

To identify the components involved in the unconventional cell-surface transport of core-glycosylated pendrin, we first examined the binding partners of H723R-pendrin using tandem affinity purification (TAP) with mass spectrometry^20^. After electrophoretic separation of proteins bound to H723R-pendrin in lysates of cells expressing H723R-pendrin alone or together with Arf1-Q71L (Fig. 3a), each protein band was purified, and protein identities were determined by liquid chromatography-tandem mass spectrometry (LC-MS/MS). Among the high Mowse-score proteins, four candidate proteins that are relevant to ERQC or protein trafficking were selected for further investigation (Supplementary Table 1). A loss-of-function study was then performed with siRNAs against each target protein (Fig. 3b–f). Knockdown (60–80%) of each target protein by siRNA treatment was confirmed using quantitative PCR (Supplementary Fig. 3a). Notably, knockdown of Hsc70 significantly decreased the surface expression of H723R-pendrin induced by Arf1-Q71L (Fig. 3e,f). In addition, BFA-induced unconventional trafficking of H723R-pendrin was diminished by treatment with siRNA targeting Hsc70 (Supplementary Fig. 3b–f). Interestingly, unconventionally surface-transported ΔF508-CFTR was also reduced by knockdown of Hsc70 (Supplementary Fig. 3g). We next examined whether overexpression of the candidate genes induced the surface expression of H723R-pendrin; however, none of the candidate proteins had this effect (Fig. 3g). These results indicate that Hsc70 is necessary, but not sufficient, for unconventional surface trafficking of H723R-pendrin.

**Figure 4:**
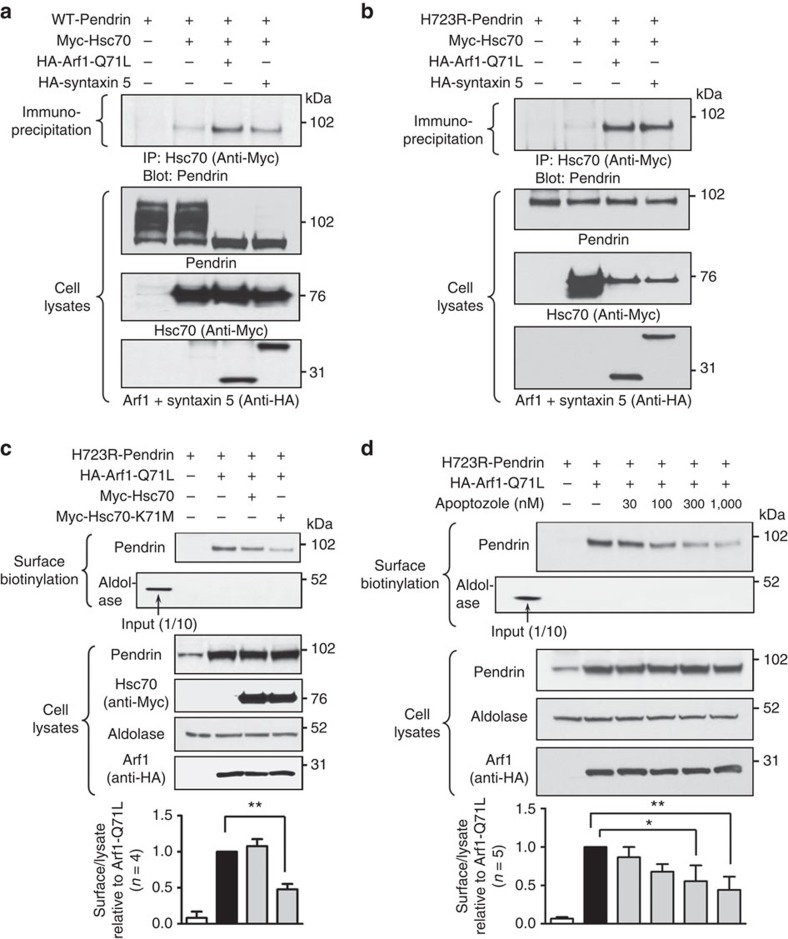
Physical interaction with Hsc70 and Hsc70 ATPase activity are necessary for unconventional surface trafficking of H723R-pendrin. (**a**,**b**) Lysates of PANC-1 cells transfected with indicated plasmids were immunoprecipitated with anti-Myc (Hsc70) and immunoblotted. Both and WT- (**a**) and H723R- (**b**) pendrins strongly interacted with Hsc70 when ER-to-Golgi traffic was blocked by Arf1-Q71L or syntaxin 5 overexpression. (**c**) Inhibition of Hsc70 ATPase activity by overexpression of Hsc70-K71M (ATPase activity-ablated dominant-negative mutant) inhibited the surface expression of unconventionally transported H723R-pendrin. (**d**) Hsc70 ATPase activity inhibitor apoptozole dose-dependently reduced Arf1-Q71L-induced surface expression of H723R-pendrin. *P<0.05, ^**^P<0.01 by one-way analysis of variance, the number of replicates (*n*) is presented in each panel. Unprocessed original scans of western blots are shown in Supplementary Fig. 10.

We then further examined the role of Hsc70 in the unconventional secretion of core-glycosylated pendrin. Immunoprecipitation results revealed that both core-glycosylated WT-pendrin (Fig. 4a) and H723R-pendrin (Fig. 4b) strongly interacted with Hsc70 when ER-to-Golgi trafficking was blocked by Arf1-Q71L or syntaxin 5 overexpression. The ATPase activity of Hsc70 plays a key role in the binding and release of Hsc70 substrates^11^. To investigate whether the ATPase activity of Hsc70 is necessary for surface transport of core-glycosylated H723R-pendrin, the ATPase-deficient Hsc70-K71M mutant^21^ was overexpressed in cells or cells were treated with apoptozole, the inhibitor of Hsc70 ATPase activity^22^. Inhibition of the ATPase activity by Hsc70-K71M reduced the unconventional surface expression of H723R-pendrin (Fig. 4c). Similarly, apoptozole inhibited the unconventional transport of H723R-pendrin in a dose-dependent manner (Fig. 4d), indicating that the ATPase activity of Hsc70 is important for the unconventional transport of immature core-glycosylated pendrin.

### DNAJC14 participates in unconventional secretion of pendrin

**Figure 5:**
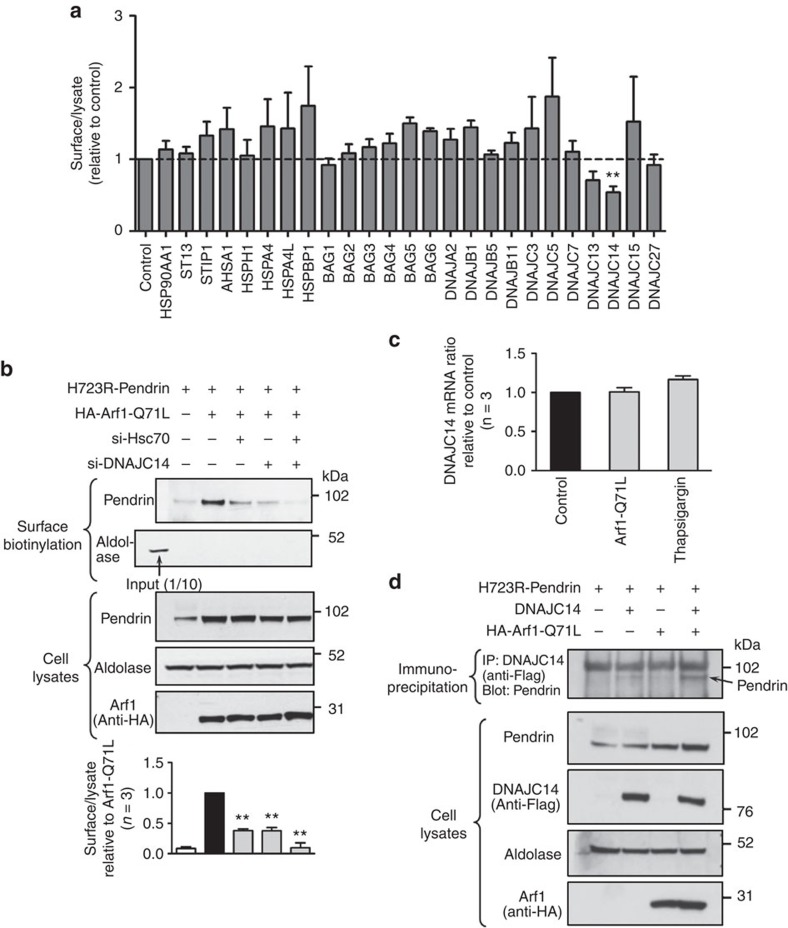
DNAJC14 is necessary for unconventional surface trafficking of H723R-pendrin. (**a**) An siRNA screen was performed in PANC-1 cells transfected with plasmids encoding H723R-pendrin and Arf1-Q71L to identify J proteins or nucleotide exchange factors involved in the unconventional transport of H723-pendrin. Cells were treated with siRNA 24 h before transfection with plasmids. Surface biotinylation assays were performed 24 h after plasmid transfection, and the surface-to-lysate ratio was calculated by densitometry. A summary of multiple experiments is shown, and representative immunoblots are presented in Supplementary Fig. 4. ^**^P<0.01 by one-way analysis of variance (ANOVA), *n*=4–7. (**b**) Treatment with siRNAs targeting DNAJC14 and Hsc70 cooperatively reduced the surface expression of unconventionally transported H723R-pendrin. ^**^P<0.01 by one-way ANOVA, difference from Arf1-Q71L alone (lane 2), *n*=3. (**c**) Quantitative PCR analyses of DNAJC14 in PANC-1 cells. Arf1-Q71L or thapsigargin did not significantly alter the mRNA levels of DNAJC14. (**d**) Proteins samples from PANC-1 cells transfected with indicated plasmids were immunoprecipitated with anti-Flag (DNAJC14) and immunoblotted. Arf1-Q71L-mediated ER-to-Golgi blockade induced the association of DNAJC14 with H723R-pendrin. The data are representative of three independent experiments. Unprocessed original scans of western blots are shown in Supplementary Fig. 10.

In the canonical model of the ATP-dependent binding and release cycle of HSP70 substrates, J proteins and nucleotide exchange factors (NEFs) are key regulators of HSP70 function^15^. In addition, co-chaperones have been suggested to be responsible for the functional diversity of the HSPs^15^. Therefore, we hypothesized that co-chaperone(s) are involved in the Hsc70-dependent unconventional trafficking of pendrin. A total of 25 J proteins, NEFs, HSP90 components, and other co-chaperones known to interact with Hsc70 were knocked down using a custom siRNA library, and Arf1-Q71L-mediated surface expression of H723R-pendrin was analysed to identify the Hsc70 co-chaperone associated with the unconventional secretion of immature core-glycosylated pendrin. The initial screening revealed that knockdown of DNAJC14 (a member of the J protein family also known as DRIP78, Jiv, or LIP6) had the strongest effect in reducing the unconventional secretion of H723R-pendrin (Fig. 5a and Supplementary Fig. 4). Subsequent analyses further confirmed the involvement of DNAJC14 in the unconventional secretion of H723R-pendrin. When Hsc70 and DNAJC14 were simultaneously depleted, the Arf1-Q71L-induced cell-surface expression of H723R-pendrin was almost completely abolished (Fig. 5b). However, DNAJC14 was not involved in the unconventional secretion of ΔF508-CFTR (Supplementary Fig. 5), implying that substrate specificity exists in the DNAJC14-mediated unconventional secretion. Quantitative PCR analyses revealed that Arf1-Q71L or thapsigargin did not affect the endogenous levels of DNAJC14 messenger RNA in PANC-1 cells (Fig. 5c). In contrast, Arf1-Q71L evoked co-immunoprecipitation between DNAJC14 and H723R-pendrin (Fig. 5d), suggesting that a physical association with the substrate protein rather than a translational regulation of DNAJC14 underlies the mechanism of DNAJC14-mediated unconventional protein secretion.

**Figure 6:**
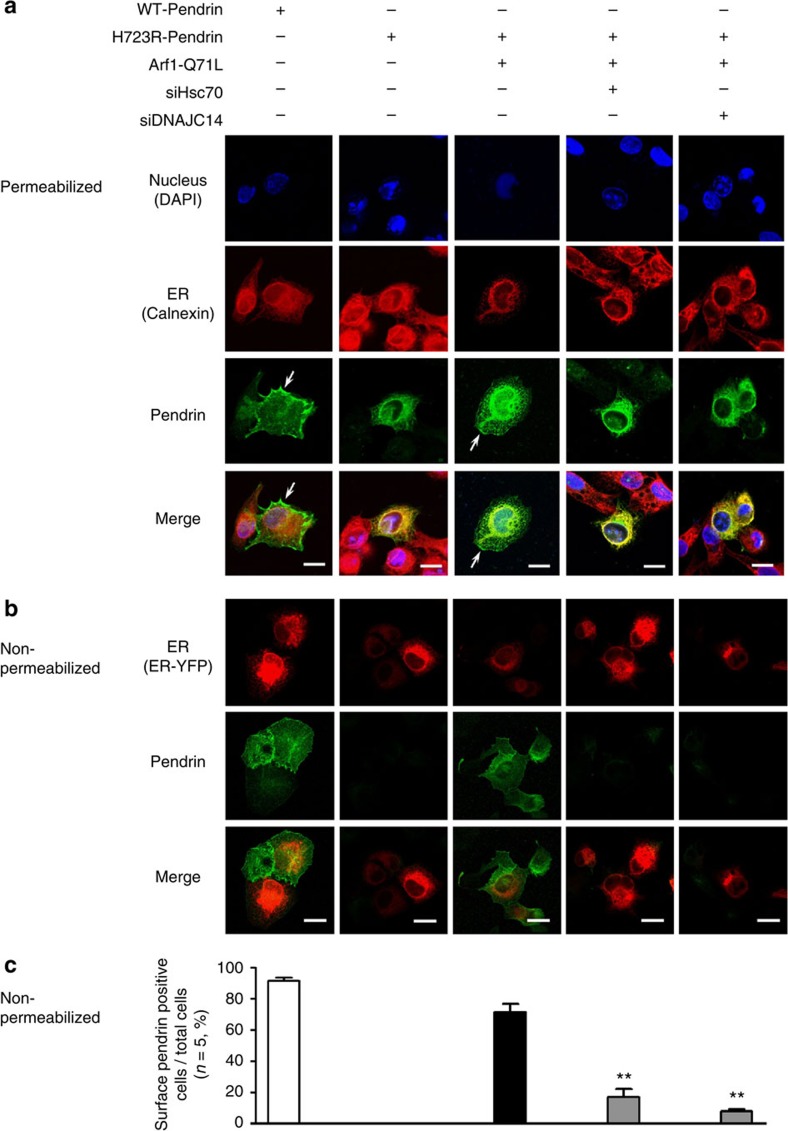
Knockdown of Hsc70 and DNAJ14 inhibits the cell-surface expression H723R-pendrin induced by Arf1-Q71L. (**a**) Cellular localization of pendrin was examined in PANC-1 cells permeabilized with ethanol and acetone. Cells stained for WT- and H723R-pendrin were co-stained for the ER marker protein calnexin. Arrows indicate the cell-surface expression of WT- or H723R-pendrin. Nuclei were counterstained with 4′,6-diamidino-2-phenylindole (DAPI). Scale bar, 20 μm. (**b**,**c**) Cell-surface expression of WT- and H723R-pendrin was examined in non-permeabilized PANC-1 cells (4% formaldehyde fixation) using antibodies against the extracellular N terminus of pendrin^8^ (Abcam, ab66702). The plasmid encoding the ER marker protein ER-yellow fluorescent protein (YFP) was co-transfected. Arf1-Q71L induced the cell-surface expression of H723R-pendrin, which was blocked by siRNAs targeting Hsc70 and DNAJC14. Quantification of the ratio of cells expressing pendrin on the cell surface relative to total cells in five independent experiments is shown in **c**. Scale bar, 20 μm. ^**^P<0.01 by one-way analysis of variance, difference from H723R-Pendrin+Arf1-Q71L alone (lane 3), *n*=5.

To further substantiate our observations, we evaluated the surface expression of pendrin using immunofluorescence analysis. In permeabilized control cells (Fig. 6a), WT-pendrin was expressed at the cell surface, whereas most H723R-pendrin was located in the ER. Notably, a significant amount of H723R-pendrin reached the cell surface when ER-to-Golgi trafficking was blocked by Arf1-Q71L. Importantly, the Arf1-Q71L-induced surface expression of H723R-pendrin was abolished by treatment with siRNA targeting Hsc70 or DNAJC14 (Fig. 6a). These events were more evident in the immunostaining of non-permeabilized cells (Fig. 6b,c). All of these biochemical and morphological results indicate that Hsc70 and DNAJC14 are necessary for the unconventional cell-surface expression of H723R-pendrin.

### Rescue of H723R-pendrin trafficking by DNAJC14 upregulation

**Figure 7:**
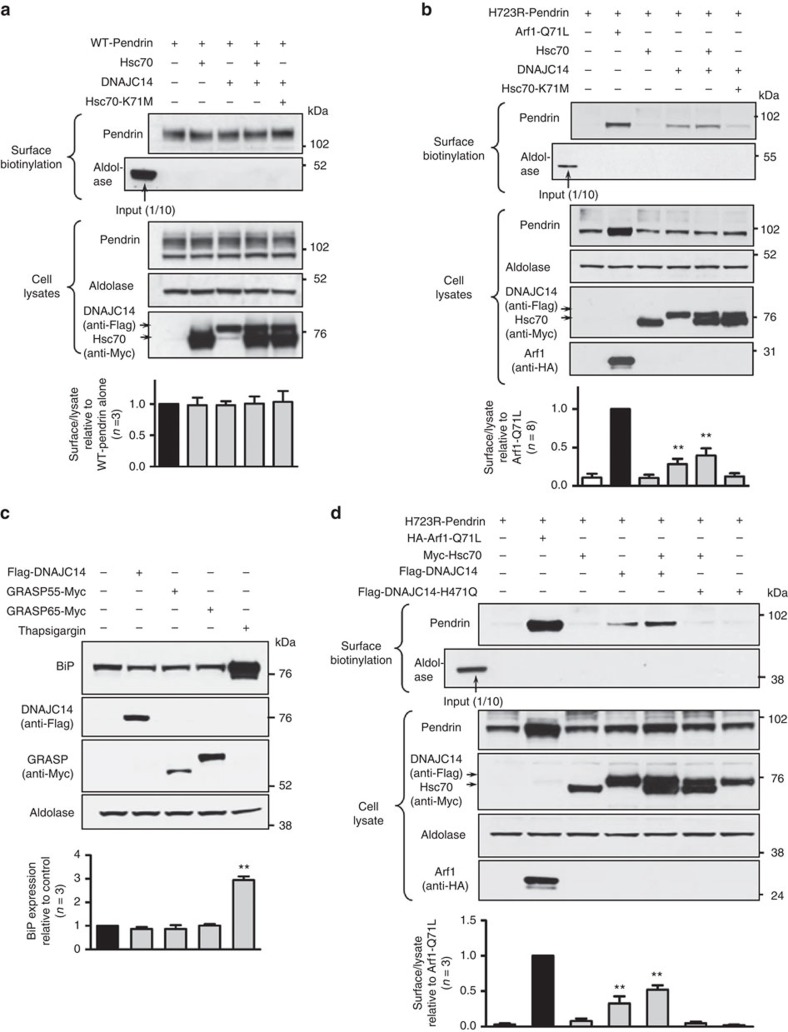
DNAJC14 upregulation restores the cell-surface expression of H723R-pendrin. (**a**) The surface expression of WT-pendrin is not affected by upregulation of Hsc70 and DNAJC14 or by inhibition of Hsc70 ATPase activity (Hsc70-K71M) in PANC-1 cells. (**b**) Overexpression of DNAJC14 alone or with Hsc70 induces the cell-surface expression of core-glycosylated H723R-pendrin in PANC-1 cells. Note that K71M abolished the effect of DNAJC14 to rescue H723R-pendrin at the surface. (**c**) Expression of BiP was analysed as an indicator of ER stress. Cells treated with thapsigargin were used as a positive control. Overexpression of DNAJC14, GRASP55 or GRASP65 did not increase BiP in PANC-1 cells. (**d**) The DNAJC14-H471Q mutation, which results in the loss of the Hsc70 ATPase accelerating activity, abolished the ability of DNAJC14 to rescue H723R-pendrin at the surface. Quantitation of multiple experiments is presented under each immunoblot. ^**^P<0.01 by one-way analysis of variance, difference from lane 1, the number of replicates (*n*) is presented in each panel. Unprocessed original scans of western blots are shown in Supplementary Fig. 10.

Next, we examined whether DNAJC14 upregulation can rescue the transport defects caused by the H723R-pendrin mutation. Control experiments with WT-pendrin indicated that neither overexpression of Hsc70 and DNAJC14 nor inhibition of Hsc70-ATPase activity by Hsc70-K71M affected the conventional surface transport of mature WT-pendrin (Fig. 7a). In contrast, overexpression of DNAJC14 alone or together with Hsc70 induced the surface expression of H723R-pendrin (Fig. 7b) without significantly inducing ER stress (Fig. 7c). Similar to the effects induced by Arf1-Q71L (Fig. 5a), DNAJC14-induced cell-surface expression of H723R-pendrin was not inhibited by the single depletion of NEFs or components of the HSP90 chaperone complex (Supplementary Fig. 6a,b). However, DNAJC14-induced surface expression of H723R-pendrin was reduced significantly by Hsc70-K71M (Fig. 7b) and by co-expression of other J proteins that may compete with DNAJC14 in associating with Hsc70 (Supplementary Fig. 6c), indicating that functionally competent Hsc70 is required for DNAJC14 activity. DNAJC14 contains a tripeptide of histidine, proline, and aspartic acid residues (HPD motif), which is highly conserved among DNAJ proteins and critical for accelerating the ATPase activity of Hsc70 (refs 21, 23). Notably, the H471Q mutation abolished the ability of DNAJC14 to induce the cell-surface expression of H723R-pendrin (Fig. 7d), further supporting the importance of the DNAJC14 and HSP70 interaction and the ATPase activity of HSP70 in the unconventional trafficking of pendrin.

**Figure 8:**
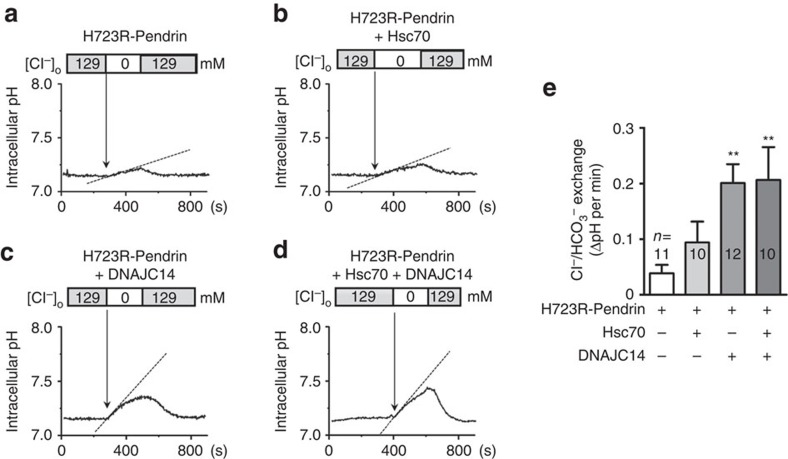
Rescue of anion exchange activity of H723R-pendrin by DNAJC14. PANC-1 cells were transfected with the indicated plasmids and Cl^−^/HCO_3_^−^ exchange activity was examined. Representative traces are shown in **a**–**d,** and a summary of multiple experiments is depicted in **e**. Overexpression of DNAJC14 alone, but not Hsc70 alone, significantly increased the Cl^−^/HCO_3_^−^ exchange activity in H723R-pendrin-expressing PANC-1 cells. ^**^P<0.01 by one-way analysis of variance, difference from H723R-pendrin alone (lane 1), the number of replicates (*n*) is presented in each lane.

Functional analyses showed that DNAJC14 alone increased the Cl^−^/HCO_3_^−^ exchange activity of H723R-expressing PANC-1 cells from 0.039±0.015 ΔpH unit min^−1^ to 0.201±0.034 ΔpH unit min^−1^ (*P*<0.01) (Fig. 8a–e). Measurement of Cl^−^/I^−^ exchange activity further confirmed that DNAJC14 upregulation induced the cell-surface expression of functionally active H723R-pendrin (Supplementary Fig. 7). Co-expression of Hsc70 did not significantly augment the DNAJC14 effects. However, Hsc70-K71M blocked the effects of DNAJC14, again indicating that the effect of DNAJC14 is dependent on the ATPase activity of endogenous Hsc70.

**Figure 9:**
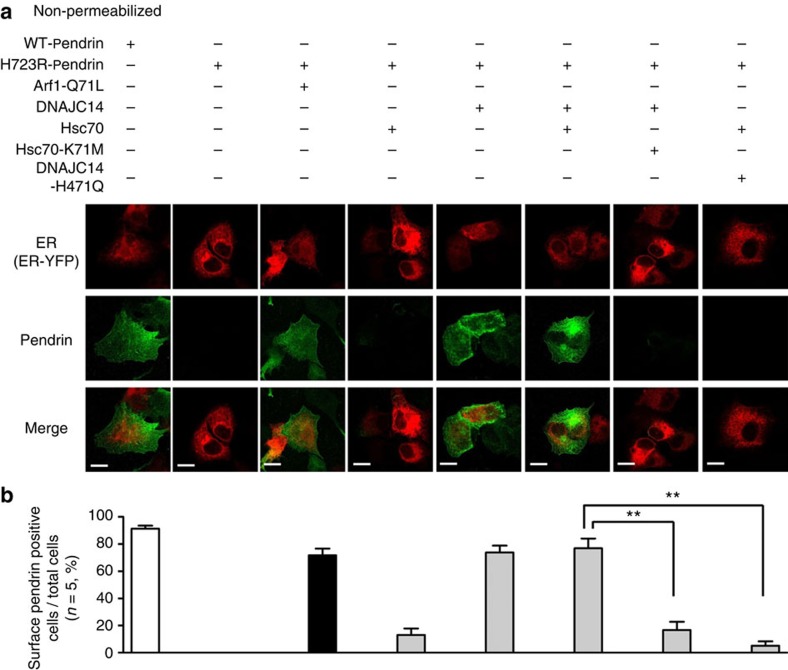
DNAJC14 restores cell-surface expression of H723R-pendrin in immunostainings. The cell-surface expression of wild-type (WT) and H723R pendrins were examined in non-permeabilized PANC-1 cells. The ER marker protein ER-YFP was co-expressed. Representative images are shown in **a.** Quantitation of results from five independent experiments is shown in **b**. Note that the Hsc70-K71M and DNAJC14-H471Q mutations strongly inhibited the ability of DNAJC14 to rescue H723R-pendrin at the cell surface. Scale bar, 20 μm. **P<0.01 by one-way analysis of variance, *n*=5.

**Figure 10:**
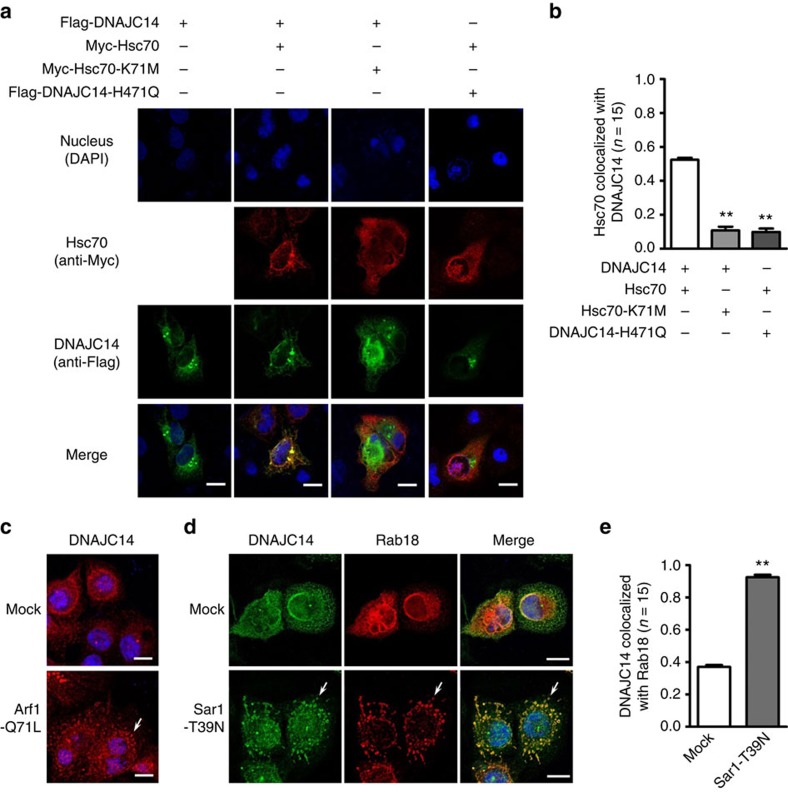
Localization of DNAJC14 and Hsc70 in PANC-1 cells. (**a**,**b**) Subcellular localization of Flag-DNAJC14 and Myc-Hsc70 was examined in permeabilized PANC-1 cells. Representative images are shown in **a**. Quantitation of results from multiple experiments is summarized in **b**. A high degree of colocalization (an average of 52%) was observed in cells stained doubly with Hsc70 and DNAJC14. The Hsc70-K71M and DNAJC14-H471Q mutations significantly reduced colocalization of Hsc70 and DNAJC14. Scale bar, 20 μm. **P<0.01 by one-way analysis of variance, difference from lane 1, *n*=15. (**c**–**e**) Endogenous DNAJC14 and Rab18 in PANC-1 cells were immunostained. ER-to-Golgi blockade by Arf1-Q71L (**c**) or Sar1-T39N (**d**) induced the movement of endogenous DNAJC14 into punctate structures and increased colocalization with Rab18 (**d**,**e**). Scale bar, 20 μm. **P<0.01 by paired Student's *t*-test, *n*=15.

The immunostaining results were consistent with the surface biotinylation and functional data. DNAJC14, alone or together with Hsc70, induced the cell-surface expression of H723R-pendrin in non-permeabilized cells. In addition, Hsc70-K71M blocked the DNAJC14-mediated effect and DNAJC14-H471Q was not effective in inducing surface trafficking of H723R-pendrin (Fig. 9a,b). Then, we examined the localization of DNAJC14 in membrane-permeabilized cells. Interestingly, although DNAJC14 is known to be an ER chaperone, overexpression of this protein led to its distribution both within the ER and in multiple cytosolic punctate structures, where it colocalized with Hsc70 (Fig. 10a,b). The Hsc70-K71M and DNAJC14-H471Q mutations significantly inhibited the colocalization of Hsc70 and DNAJC14 (Fig. 10a,b). In an effort to identify the punctate structures, cells were immunostained for multiple markers of organelles and vesicles. Of these, overexpressed DNAJC14 clearly colocalized with Rab18, which is considered to be a marker of lipid droplets^24^ (Supplementary Fig. 8). The association between DNAJC14 and Rab18-containing structures was also recapitulated on examination of endogenous DNAJC14. In control mock-transfected PANC-1 cells, endogenous DNAJC14 was more evenly distributed in the cells and only a small fraction colocalized with Rab18 in perinuclear areas. Remarkably, ER-to-Golgi blockade by Arf1-Q71L (Fig. 10c) or Sar1-T39N (Fig. 10d) induced the movement of endogenous DNAJC14 into the punctate structures where Rab18 was also enriched (Fig. 10d,e).

## Discussion

Most signal peptide-containing proteins, including transmembrane proteins, are synthesized in the ER and reach the plasma membrane by following the well-documented Golgi-mediated secretion pathway. However, recent evidence suggests that many cytosolic, nuclear, secretory and membrane proteins reach the cell surface by unconventional secretion pathways^25^. The unusual secretion events were first recognized by the release of cytoplasmic proteins that lack a signal peptide, such as FGF2 and interleukin 1β, into the extracellular space. Then, it was observed that signal peptide-containing proteins are also delivered to the plasma membrane via a route that bypasses the Golgi apparatus. In general, it is believed that most unconventional secretion events are not constitutive but stress-induced^26^. Cellular stresses, such as inflammation, starvation and mechanical stress during development, induce unconventional secretion of cytoplasmic and membrane proteins. However, mechanisms and molecular components of these pathways are still not fully understood. In the present study, we provide evidence that Hsc70 and DNAJC14 are involved in the unconventional secretion pathway induced by ER stress. Under normal conditions, transmembrane proteins that pass ERQC are delivered to the plasma membrane via the conventional Golgi-mediated route, and proteins that do not pass ERQC undergo ERAD. During ER stress or ER-to-Golgi blockade, unfolded protein response is activated to recruit, synthesize and cluster many ER and cytosolic chaperones necessary for the removal of accumulated protein in the ER. The activation of chaperone machinery in turn stimulates the activation of the unconventional secretion pathway, in addition to the well-established ERAD pathway, to relieve the protein burden in the ER. In this process, the ER co-chaperone DNAJC14, in cooperation with the cytosolic chaperone Hsc70, plays a critical role in targeting the transmembrane protein for unconventional secretion.

Hsc70 is a constitutively expressed molecular chaperone and achieves functional diversity based on specific cooperation with co-chaperones. Hsc70 facilitates biogenesis and stabilization of CFTR in cooperation with Hdj1 (DNAJB1), Hdj2 (DNAJA1) and HOP (Hsp-organizing protein)^13^,^27^,^28^, whereas it promotes protein ubiquitination and proteasomal degradation of misfolded CFTR when present with CHIP in eukaryotic cytosol^14^. In addition to protein folding and degradation, Hsc70 has been shown to be involved in the trafficking of proteins and small molecules, such as the uncoating of clathrin-coated pits, the recycling and endocytosis of synaptic vesicles for neurotransmitter release, and the maturation of steroid receptors^29^,^30^,^31^,^32^.

An important finding of the present study is the novel role of DNAJC14 in regulating the function of Hsc70. DNAJC14 was known to modulate the cell-surface trafficking of dopamine D1 receptor, the SNARE complex-mediated lysosomal trafficking, and the specific membrane targeting of yellow fever virus replication proteins^24^,^33^,^34^. However, the global role of DNAJC14 as a J domain-containing HSP70 co-chaperone remains elusive. The present results suggest that DNAJC14 switches the function of Hsc70 to divert the substrate protein (for example, H723R-pendrin) from proteasomal degradation to cell-surface secretion. DNAJC14 is an ER membrane protein, but both the NH_2_ and COOH termini and major functional domains, including the J domain, are exposed to the cytosol^24^. These structural characteristics imply that transmembrane proteins synthesized in the ER may be substrates of DNAJC14 during ER-to-Golgi blockade or ER stress conditions. The H723 residue of pendrin is located in the STAS (sulfate transporter and anti-sigma factor antagonist) domain of pendrin, which faces the cytosol. Under resting conditions, the cytosolic chaperone Hsc70 and its co-chaperones would censor the folding defects induced by the H723R mutation and bind to the misfolded pendrin to correct the folding defects. However, under conditions of DNAJC14 upregulation, such as during ER stress or in cells overexpressing exogenous DNAJC14, the J protein DNAJC14 may first bind to the client protein of folding-deficient H723R-pendrin, according to the canonical model of HSP70 chaperone machinery^15^, and bring it to the ATP-bound form of Hsc70. Then, the J domain-mediated stimulation of ATPase activity would convert Hsc70 into an ADP-bound form, and this process would stabilize the interaction with the client cargo protein. As shown in Figs 4, 7, 9 and 10, the ATPase activity of Hsc70 is essential for the DNAJC14-mediated exodus of H723R-pendrin from ERAD to unconventional secretion. HSP70s invariably require a J protein and, almost always, an NEF as partners^15^. While a specific J protein (DNAJC14) is required for the unconventional secretion of pendrin, depletion of any individual NEF was not critical for the cell-surface expression of H723R-pendrin induced by Arf1-Q71L (Fig. 5a) or by upregulation of DNAJC14 (Supplementary Fig. 6a,b). These results suggest that a redundancy exists among the functions of NEF isoforms and that they are not critically involved in the substrate specificity of Hsc70-mediated unconventional secretion.

Interestingly, conditions that induced unconventional cell-surface expression of CFTR and pendrin did not evoke unconventional secretion of the transferrin receptor (Supplementary Fig. 9), indicating that not all membrane proteins are unconventionally delivered to the cell surface during ER-to-Golgi blockade or ER stress. Furthermore, although IRE1 and Hsc70 are commonly involved in the unconventional secretion of CFTR and pendrin (Supplementary Figs 1 and 3), there are also significant differences between the unconventional pathways of CFTR and pendrin (Fig. 1; Supplementary Figs 2 and 5). Taken together, these results suggest that a certain level of substrate specificity exists in ER stress-associated unconventional secretion, in particular, in the cargo recognition step (for example, CFTR by GRASPs and pendrin by DNAJC14). In the case of GRASP55, phosphorylation of its C terminus by an IRE1-mediated signal is critical for association with the cargo protein CFTR^17^. Although the results in Fig. 5 indicate that a physical association with DNAJC14 underlies the Arf1-Q71L-induced unconventional secretion of pendrin, the precise molecular mechanism needs to be determined in future studies. It will be an intriguing task to investigate whether phosphorylation(s) at specific loci of DNAJC14 is required for ER-to-Golgi blockade-induced unconventional secretion of pendrin.

Blockade of the ER-to-Golgi transport induces an accumulation of many secretory and ER-resident proteins in the ER lumen^17^. This, in turn, will saturate the ERAD capacity of the cells and increase the total cellular levels of H723R-pendrin. In addition, activated DNAJC14 competes with other J proteins that would be involved in the degradation of misfolded proteins (Supplementary Fig. 6c), which may further contribute to the increase in the total protein level of H723R-pendrin. In fact, the results in Fig. 1c–e indicate that Arf1-Q71L increased H723R-pendrin in cell lysates by an average of 136±29% (*n*=11). These findings may raise the possibility that accumulation of H723R-pendrin in the ER evokes nonspecific leakage of protein to the cell surface. However, several lines of evidence indicate that this is not the case and that a specific regulatory pathway is involved in the ER-to-Golgi blockade-induced cell-surface expression of H723R-pendrin. First, inhibition of Hsc70 or DNAJC14, which did not affect the cytosolic levels of H723R-pendrin, abolished the unconventional secretion of H723R-pendrin (Fig. 5b). Second, exogenous expression of DNAJC14, which did not increase cytosolic H723R-pendrin, evoked the cell-surface expression of H723R-pendrin (Fig. 7b). Last, all quantitative analyses of surface biotinylation assays using a ratio of surface/total pendrin indicated that ER-to-Golgi blockade or induction of ER stress prompted much higher cell-surface expression of core-glycosylated pendrin than its cytosolic increase, robustly suggesting the unconventional secretion of pendrin.

A remaining question is the nature of the vesicular traffic system that mediates the unconventional secretion of membrane protein cargos. The Rab18-containing vesicles seem to be responsible for the early secretion pathway immediately after ER exit as DNAJC14 was colocalized at these structures (Fig. 10c–e; Supplementary Fig. 8). Rab18 is the Ras-related small GTPase and is related to lipid droplet formation from the ER membrane^35^,^36^. Interestingly, the membrane protein caveolin-1 has been shown to be redirected from the ER to lipid droplet vesicles under certain ER stress conditions^37^. Lipid droplets are, in general, composed of phospholipid monolayers. However, certain specific sites, such as ‘wrinkles', would create ridges with local bilayer properties to which membrane proteins, such as class I major histocompatibility complex, could be anchored^38^. These findings suggest that lipid droplet vesicles might be involved in unconventional secretory transit of transmembrane proteins. However, there is also the possibility that vesicular traffics other than lipid droplets mediate the unconventional secretion. Future studies investigating the downstream trafficking machinery should aid in understanding the big picture of unconventional protein secretion.

Defects in protein folding can induce two types of diseases in human^11^. The first group is caused by the accumulation of aberrant proteins and its toxic effects, such as Huntington's, Parkinson's and Alzheimer's diseases. It is also plausible that disposal of misfolded proteins through the activation of unconventional secretion would be beneficial in treating patients with diseases caused by the toxic intracellular aggregation of misfolded proteins. The second group of diseases is characterized by the premature degradation of misfolded proteins, which eliminates the original function of the protein, such as in cystic fibrosis and Pendred syndrome. Although they have some defects in protein folding, many of these prematurely degraded membrane proteins, such as ΔF508-CFTR and H723R-pendrin, retain a certain level of their original activity when they reach the cell surface^10^,^17^,^39^. Therefore, many pharmacological and molecular efforts have been invested in approaches to facilitate the membrane targeting of these proteins. In this study, we showed that functionally active H723R-pendrin can be successfully transported to the epithelial cell surface by targeting it to unconventional protein secretion. The selective activation of this pathway may be a promising drug target for the treatment of patients with congenital deafness caused by folding-deficient pendrin mutants. Considering that DFNB4 and Pendred syndrome are progressive until adolescence^40^,^41^, earlier diagnosis and correction would be beneficial for these patients. However, there would be some hurdles to overcome before a final implementation of this strategy in the clinic. For example, the cell-surface transported folding-deficient membrane proteins remain under the control of peripheral protein quality control, and thus, their retention time at the plasma membrane is relatively short^42^. A strategy combined with agents that inhibit peripheral quality control would be a measure to overcome this problem.

## Methods

### Cell culture and transfection

PANC-1 and HEK 293 cells were maintained in Dulbecco's modified Eagle medium-HG (Invitrogen, Carlsbad, CA, USA) supplemented with 10% fetal bovine serum and penicillin (50 IU ml^−1^)/streptomycin (50 μg ml^−1^). The mammalian expression plasmids for WT- and H723R-pendrin were subcloned into pcDNA3.1(+) (Invitrogen) and TAP expression vectors (Stratagene, La Jolla, CA, USA) from pCMV-myc-human pendrin described previously^10^, using the KpnI (5′)/NotI (3′) and BamHI(5′)/XhoI(3′) sites, respectively. Plasmids encoding human CFTR (WT-CFTR and ΔF508-CFTR), GRASP55, HA-STX5, Myc-Sar1 and HA-Arf1 were described previously^17^. Human Hsc70, NckAP1, KIAA0980 and DNAJC14 clones were purchased from Open Biosystems (Lafayette, CO, USA) and subcloned into pCMV-myc or pCMV-Flag plasmids (Clontech, Palo Alto, CA, USA). Human Rab18-HA clone was purchased from Sino Biological (Beijing, China). pEYFP-ER was purchased from BD Biosciences (San Jose, CA, USA). Mutant plasmids were generated with a PCR-based site-directed mutagenesis kit (Stratagene), according to the manufacturer's protocol. Oligonucleotide primer sequences for complementary DNA subcloning and mutagenesis are presented in Supplementary Table 2. A customized siRNA library (ON-TARGETplus as SMARTpool reagent) was purchased from Dharmacon (Lafayette). Detailed information for siRNAs used in this study is summarized in Supplementary Table 3. Plasmids or siRNAs were transiently transfected into the cells using Lipofectamine 2000 reagent (Invitrogen).

### Chemicals and antibodies

Thapsigargin and BFA were purchased from Sigma-Aldrich (St Louis, MO, USA). Apoptozole was purchased from Millipore (Billerica, MA, USA). Anti-pendrin (G-19, epitope against C terminus), anti-HA, anti-Myc, anti-aldolase and anti-Flag antibodies were from Santa Cruz Biotechnology (Santa Cruz, CA, USA); anti-pendrin (ab66702, epitope against N terminus), anti-calnexin, anti-giantin, anti-Lamp1, anti-CHMP2B, anti-ATG7 and anti-Hsc70 were from Abcam (Cambridge, UK); anti-EEA1 was from BD Biosciences; anti-CD9 was from LSBio (Seattle, WA, USA); anti-DNAJC14 was from Novus (Littleton, CO, USA); anti-Rab7 was from Cell Signaling Technologies (Danvers, MA, USA); and anti-CFTR antibody (M3A7) was from Millipore. Detailed information for primary and secondary antibodies used in this study is summarized in Supplementary Table 4.

### Immunoprecipitation and surface biotinylation assay

For immunoprecipitation, cell lysates were mixed with the appropriate antibodies and incubated overnight at 4 °C in a lysis buffer containing 50 mM Tris-HCl (pH 8.0), 150 mM NaCl, 1% (v/v) Triton X-100, and complete protease inhibitor mixture (Roche Applied Science, Mannheim, Germany). Immune complexes were precipitated using mixed protein A/G beads (Pierce, Rockford, IL, USA) and then washed three times with lysis buffer before elution with 2 × sodium dodecyl sulfate (SDS) sample buffer. Protein samples were separated by SDS-polyacrylamide gel electrophoresis (PAGE). The separated proteins were transferred to a nitrocellulose membrane and blotted with appropriate primary and secondary antibodies. Protein bands were detected by enhanced chemiluminescence, and the densities of the bands were measured using imaging software (Multi Gauge ver. 3.0; Fujifilm, Tokyo, Japan). For surface biotinylation assay, the surface proteins were biotinylated with sulfo-NHS-SS-biotin (Pierce) for 30 min before cell lysis. Cells were washed with quenching buffer containing 1% bovine serum albumin (BSA) and then washed three times with phosphate-buffered saline (PBS). After cell lysis, the lysates were incubated overnight at 4 °C with 10% NeutrAvidin beads (Pierce). NeutrAvidin-bound biotinylated proteins were centrifuged and washed three times with PBS and then eluted in 2 × sample buffer. The next steps of the biotinylation assay were identical to those for the immunoblotting procedure described above. For the digestion of glycosylated pendrin by Peptide-*N*-glycosidase F (PNGase F; New England Biolabs, Ipswich, MA, USA), protein samples were incubated with PNGase F (500 U per reaction) in solutions containing 1% Triton X-100 for 2 h.

### TAP and identification of proteins by LC-MS/MS

TAP was performed using TAP expression vector with two tandem affinity tags, a streptavidin-binding peptide and a calmodulin-binding peptide (Stratagene). Cell lysates were applied to streptavidin resin, and unbound proteins were washed three times with streptavidin-binding buffer according to the manufacturer's protocol. Eluted protein complexes were applied to calmodulin resin and contaminants were removed by washing them three times with calmodulin-binding buffer. Tagged proteins and interacting partners were eluted with elution buffer, and eluted proteins were separated by SDS–PAGE. Protein bands of interest were excised and digested with trypsin using the following protocol^43^. Each protein band was excised from the PAGE gel, placed in a polypropylene tube, and briefly washed 4–5 times with 150 μl 1:1 acetonitrile (ACN) per 25 mM ammonium bicarbonate (ABC), pH 7.8. The gel slices were dehydrated in 100% ACN and then dried in a Speedvac concentrator. After reduction and alkylation, the gel slices were rehydrated in 30 μl 25 mM ABC containing 20 μg trypsin. After incubation at 37 °C for 20 h, the liquid was transferred to a clean tube. Tryptic peptides remaining in the gel matrix were extracted for 40 min at 30 °C with 20 μl 50% (v/v) aqueous ACN solution containing 0.1% (v/v) formic acid. The combined supernatants were evaporated in the Speedvac and dissolved in 8 μl 5% (v/v) aqueous ACN containing 0.1% (v/v) formic acid for mass spectrometric analysis. The resulting tryptic peptides were separated and analysed using a reverse phase capillary high-pressure liquid chromatography column directly coupled to a Finnigan LCQ ion-trap mass spectrometer (LC-MS/MS)^44^. Both the 0.1 × 20-mm trapping and the 0.075 × 130-mm resolving columns were packed with Vydac 218MS low trifluoroacetic acid C18 beads (5 μm diameter, 300 Å pore size) and placed in-line. The peptides were bound to the trapping column for 10 min in 5% (v/v) aqueous ACN containing 0.1% (v/v) formic acid, and the bound peptides were eluted with a 50-min gradient of 5–80% (v/v) ACN containing 0.1% (v/v) formic acid at a flow rate of 0.2 μl min^−1^. For tandem mass spectrometry, the full mass scan range mode was an *m*/*z* of 450–2,000 Da. After determination of the charge states of an ion using zoom scans, product ion spectra were acquired in MS/MS mode with a relative collision energy of 55%. The individual MS/MS spectra were processed using the TurboSEQUEST software (Thermo Quest, San Jose, CA, USA). The generated peak list files were used to query either the MSDB or NCBI database using the MASCOT program (http://www.matrixscience.com). Modifications of methionine and cysteine (carbamidomethyl [C], deamidated [NQ], oxidation [M]) with a peptide mass tolerance of 2 Da, MS/MS ion mass tolerance of 1 Da, allowance of missed cleavage of 1, and charge states of +1, +2, and +3, were all taken into account. Only significant hits as defined by MASCOT probability analysis were considered initially.

### Immunocytochemistry

PANC-1 cells were cultured on coverslips and immunostained 1 day after transfection. Cells grown on coverslips were fixed and permeabilized by incubation in cold ethanol and acetone (1:1) for 8 min at −20 °C. Nonspecific binding sites were blocked by incubation with 0.2 ml PBS containing 5% horse serum, 1% BSA and 0.1% gelatin (blocking medium) for 1 h at room temperature. After the blocking step, cells were stained by incubating with appropriate primary antibodies and then treated with fluorophore-tagged secondary antibodies. Some cells were treated with primary and secondary antibodies after fixation in 4% formaldehyde without permeabilization to stain pendrin on the cell surface. Fluorescence images were obtained with a Zeiss LSM 780 confocal microscope (Carl Zeiss, Berlin, Germany). Morphometric analysis of the captured confocal images was performed using the MetaMorph microscopy analysis software (version 7.1; Molecular Devices, Sunnyvale, USA). For the quantification of the images under each condition, 24-bit confocal images containing red, green and blue components were converted into three 8-bit mono-channel images. The colocalization of DNAJC14 and each marker protein was analysed using the colocalization module of the MetaMorph Apps package. For the analyses of the surface expression of pendrin in non-permeabilized cells, pixels above the threshold level of 30 were defined as surface pendrin positive.

### Quantitative real-time PCR

Quantitative real-time PCR (qPCR) was performed to examine the efficiency of siRNA treatments (Supplementary Fig. 3a) and to quantify DNAJC14 mRNA (Fig. 5b). qPCR primers for NckAP1 (Bioneer, Daejeon, Korea; catalogue no. P157442), Hsc70 (Bioneer, P219307), AP3B1 (Bioneer, P132135) and KIAA0980 (Bioneer, P257561) and a TaqMan probe for DNAJC14 (Applied Biosystems, catalogue no. Hs00372873_m1) were from commercial sources. Purified RNA samples from PANC-1 cells were reverse-transcribed using the iScript Select cDNA Synthesis Kit (Bio-Rad, Hercules, CA, USA). The total reaction volume was adjusted to 25 μl with RNase-free water after mixing 500 ng cDNA, 1 μl primer (or 1 μl TaqMan probe for DNAJC14) and 12.5 μl 2 × qPCR SYBR Master Mix (Applied Biosystems, catalogue no. 4309155; or 12.5 μl 2 × TaqMan Gene Expression Master Mix for DNAJC14, Applied Biosystems, catalogue no. 4369016). Amplification was performed under the following cycling conditions: 95 °C for 15 min followed by 40 cycles of 95 °C for 15 s and 60 °C for 40 s. Triplicate analyses were performed for each cDNA. Relative mRNA expression levels were calculated via the comparative threshold cycle (C_t_) method with GAPDH as the control as follows: ΔC_t_=C_t_(GAPDH)−C_t_(target gene). The fold-change in gene expression normalized to GAPDH and relative to the control sample was calculated as 2^−^ΔΔ^Ct^.

### Measurement of pH_i_ and Cl^−/^HCO_3_^−^ exchange activity

Measurements of pH_i_ in HEK 293 and PANC-1 cells were performed with the pH-sensitive fluorescent probe 2′,7′-bis-(2-carboxyethyl)-5-(and-6)-carboxyfluorescein (BCECF). After a 24–48-h transfection with plasmids encoding WT or H723R pendrin, cells were incubated with 2 μM BCECF acetoxy-methylester for 5 min and then perfused with a HCO_3_^−^-buffered solution (120 NaCl, 5 KCl, 1 MgCl_2_, 1 CaCl_2_, 10 D-glucose, 5 HEPES and 25 NaHCO_3_ in mmol l^−1^, pH 7.4). BCECF fluorescence was recorded at the excitation wavelengths of 490 and 440 nm at a resolution of 2/ s^−1^ on a recording setup (Delta Ram; PTI Inc., Edison, New Jersey, USA). Cl^−^/HCO_3_^−^ exchange activities were estimated from the initial rate of pH_i_ increase as a result of Cl^−^ removal in the HCO_3_^−^-containing buffer (25 mM HCO_3_^−^ with 5% CO_2_). pH_i_ calibration was done with standard pH solutions containing 150 mM KCl and 5 μM nigericin (Sigma-Aldrich). The intrinsic buffer capacity (β_i_) was calculated by measuring ΔpH_i_ in response to 5–40 mM NH_4_Cl pulses in Na^+^-free solutions. Because the β_i_ values were not significantly affected by transfection with plasmids encoding WT-pendrin, H723R-pendrin, Hsc70 or DNAJC14, the Cl^−^/HCO_3_^−^ exchange activities were expressed as ΔpH unit min^−1^ without compensating for the buffer capacity.

### Measurement of Cl^−^/I^−^ exchange activity of pendrin

Cl^−^/I^−^ exchange activity of pendrin was measured with the halide-sensitive fluorescent sensor yellow fluorescent protein (YFP)-H148Q/I152L/F46L (ref. 45). Fluorescence was detected using a FLUOstar fluorescence plate reader (BMG Labtech, Durham, North Carolina, USA) with excitation (485±12 nm) and emission (>520 nm) filters. Cells in a 96-well plate were washed three times with 150 mM NaCl solution, leaving 50 μl in each well. The plate was transferred to a plate reader and the initial fluorescence was recorded for 20 s (400 ms per point). Then, 150 μl of 150 mM NaI solution was added to each well. The I^−^ influx mediated by the Cl^−^/I^−^ exchange activity of pendrin quenched the fluorescence of YFP, and the slope of fluorescence change was analysed.

### Statistical analysis

The results of the multiple experiments are presented as mean±s.e.m. Statistical analysis was performed using either Student's *t-*tests or an analysis of variance followed by the Newman–Keuls multiple comparison test, where appropriate. *P*<0.05 was considered statistically significant.

## Additional information

**How to cite this article:** Jung, J. *et al*. The HSP70 co-chaperone DNAJC14 targets misfolded pendrin for unconventional protein secretion. *Nat. Commun.* 7:11386 doi: 10.1038/ncomms11386 (2016).

## Supplementary Material

Supplementary Information Supplementary Figures 1-10 and Supplementary Table 1-4
