# Supplementary material for: The HSP70 co-chaperone DNAJC14 targets misfolded pendrin for unconventional protein secretion
Source: Nat Commun. 2016 Apr 25;7:11386. doi: 10.1038/ncomms11386 (PMC4848490; doi:10.1038/ncomms11386)
Supplement: Supplementary Information — Supplementary Figures 1-10 and Supplementary Table 1-4 [file ncomms11386-s1.pdf]

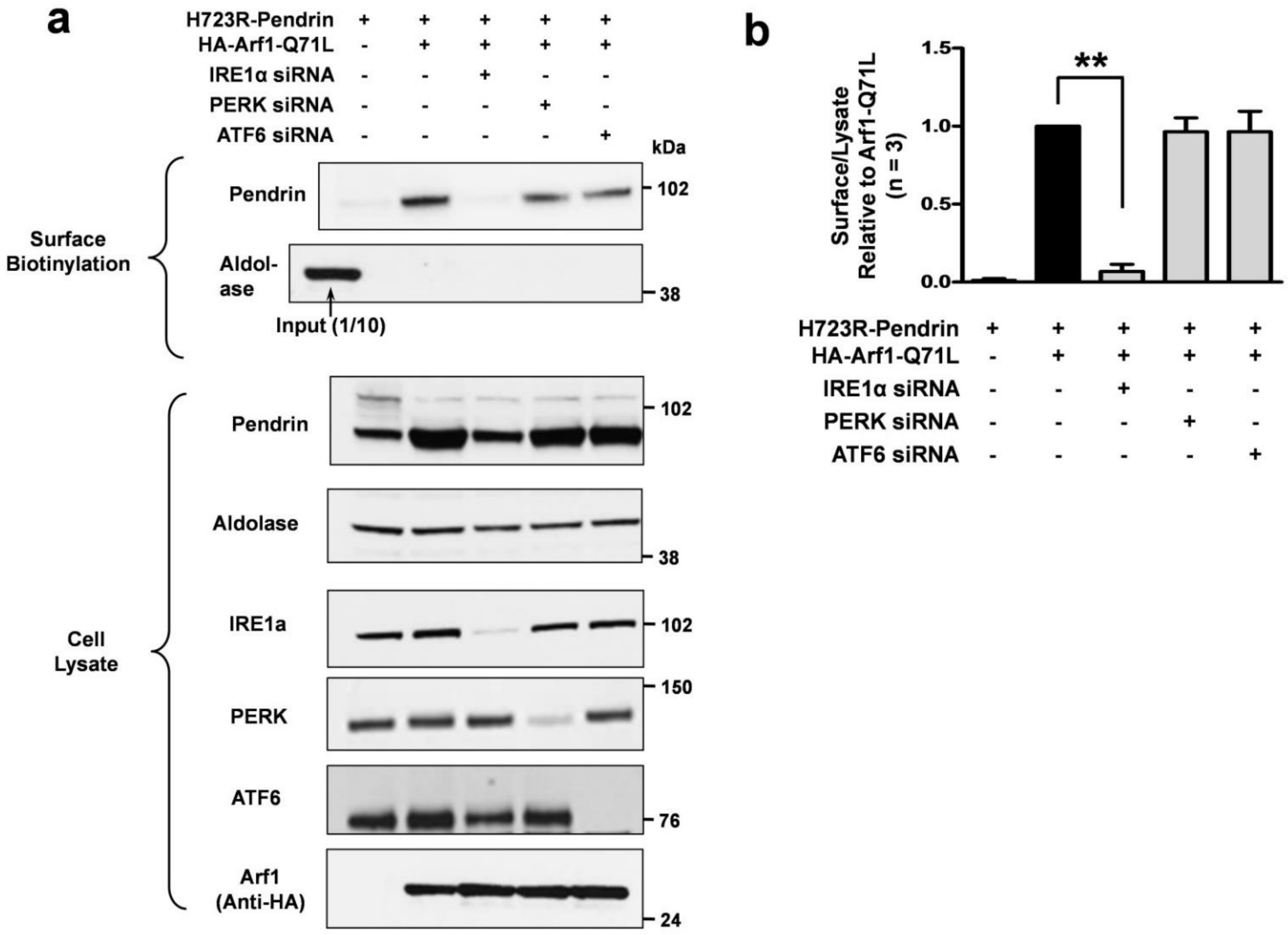

Supplementary Figure 1 | IRE1 is involved in the Arf1-Q71L-induced cell-surface expression of H723R-pendrin. PANC-1 cells were treated with siRNAs targeting IRE1α, PERK, and ATF6 at 24 h before transfection with plasmids expressing H723R-pendrin and Arf1-Q71L. Surface biotinylation assays were performed 24 h after plasmid transfection. Representative immunoblot images are shown in **a**. Quantitation of multiple experiments is presented in **b**. Depletion of IRE1α strongly inhibited Arf1-Q71L-induced cell-surface expression of H723R-pendrin. Other legends are the same as shown in Figure 1. ** $P < 0.01$ by one-way ANOVA, difference from Arf1-Q71L alone (lane 2), $n = 3$. Unprocessed original scans of western blots are shown in Supplementary Fig. 10.

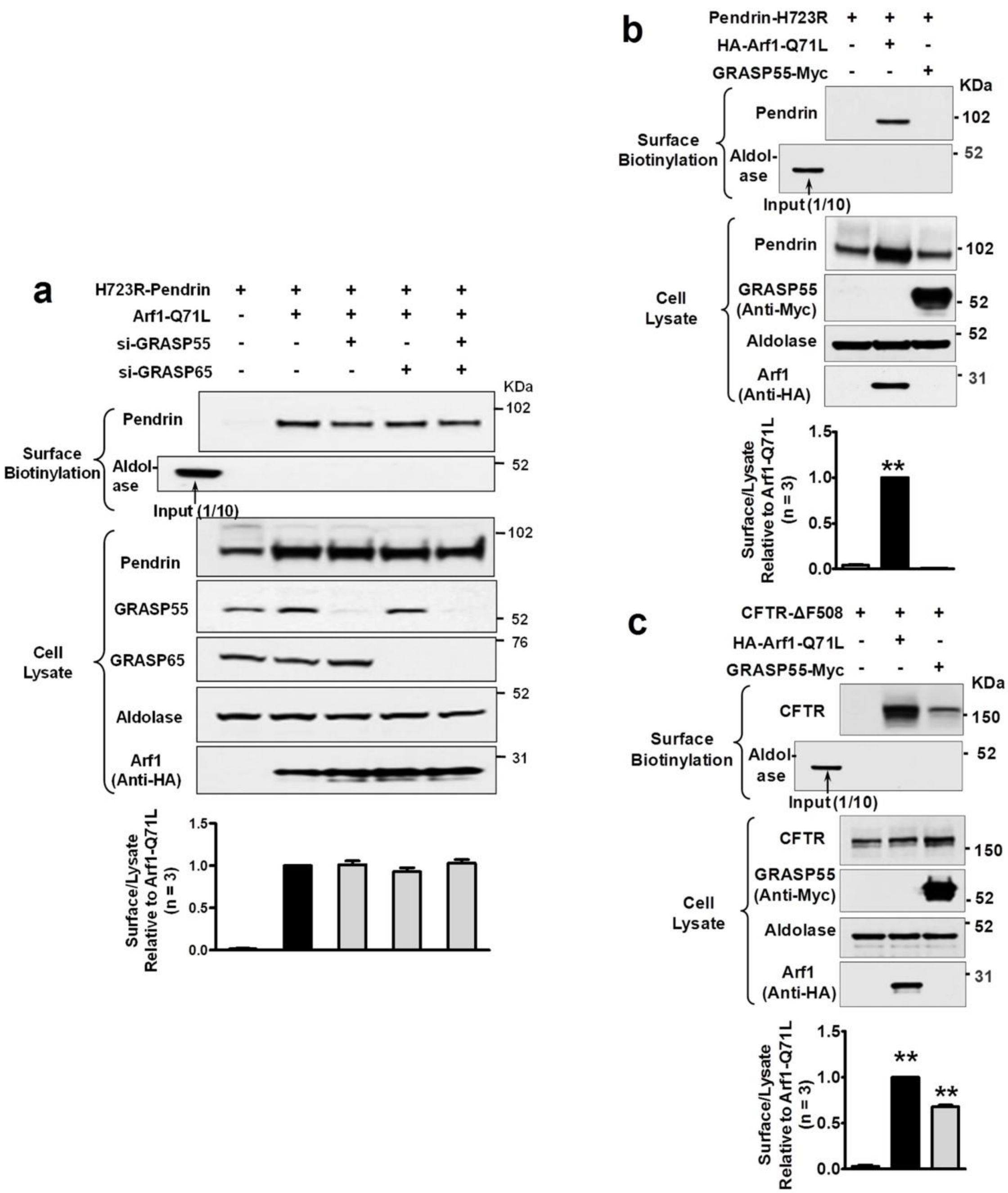

Supplementary Figure 2 | GRASPs are not involved in the Arf1-Q71L-induced cell-surface expression of H723R-pendrin. (**a**) PANC-1 cells were treated with siRNAs targeting GRASP55 and GRASP65 at 24 h before transfection with plasmids expressing H723R-pendrin and Arf1-Q71L. Surface biotinylation assays were performed 24 h after plasmid transfection. Depletion of neither GRASP55 nor GRASP65 inhibited the Arf1-Q71L-induced cell-surface expression of H723R-pendrin. (**b**, **c**) Effects of GRASP55 overexpression were re-examined using a C-terminal Myc-tagged GRASP55 plasmid in order to clearly visualize the expression of exogenous GRASP55. PANC-1 cells were transfected with the indicated plasmids and surface biotinylation assays were performed. Similar to the results obtained with non-tagged GRASP55 plasmids (Fig. 1e, f), GRASP55 was not effective in inducing cell-surface expression of H723R-pendrin (**b**), while it induced the cell-surface expression of ΔF508-CFTR (**c**). Quantitation of multiple experiments is presented under each immunoblot. $^{**}P < 0.01$ by one-way ANOVA, compared to lane 1, the number of replicates (*n*) is presented in each panel. Unprocessed original scans of western blots are shown in Supplementary Fig. 10.

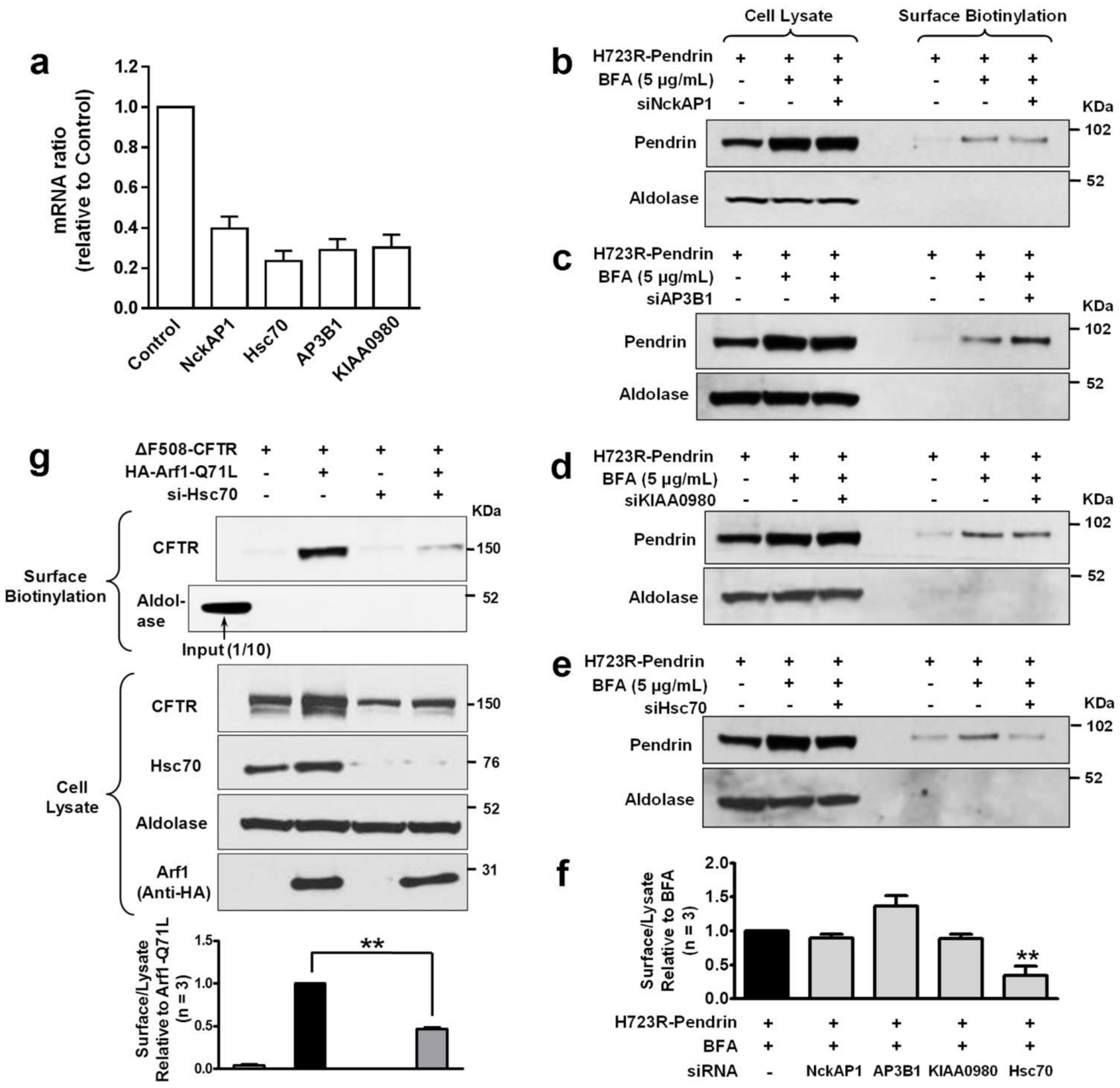

Supplementary Figure 3 | Effects of treatment with siRNAs targeting binding partners of H723R-pendrin. (**a**) Efficiency of each siRNA treatment was validated by quantitative real-time PCR. Control cells were treated with scrambled siRNA. Cells were treated with each siRNA (50 nM) for 48 h. The fold-change in gene expression normalized to GAPDH and relative to the control sample was calculated using the $2^{-\Delta\Delta Ct}$ method (n = 4). (**b-f**) To induce the unconventional cell-surface expression of H723R-pendrin, PANC-1 cells were treated with brefeldin A (BFA). Then, surface biotinylation assays were performed after knockdown of each candidate protein identified in Supplementary Table 1. Representative immunoblot images are shown in **b-e**. Quantitation of multiple experiments is presented in **f**. siRNA targeting Hsc70 reduced BFA-induced cell-surface expression of H723R-pendrin. ** $P < 0.01$ by one-way ANOVA, $n = 3$. (**g**) Treatment with siRNA against Hsc70 inhibited the Arf1-Q71L–induced unconventional cell-surface expression of ΔF508-CFTR in PANC-1 cells. siRNA-mediated knockdown of endogenous Hsc70 was visualized in the immunoblot using anti-Hsc70. Quantitation of multiple experiments is presented under the immunoblot images. ** $P < 0.01$ by one-way ANOVA, difference from lane 1, $n = 3$. Unprocessed original scans of western blots are shown in Supplementary Fig. 10.

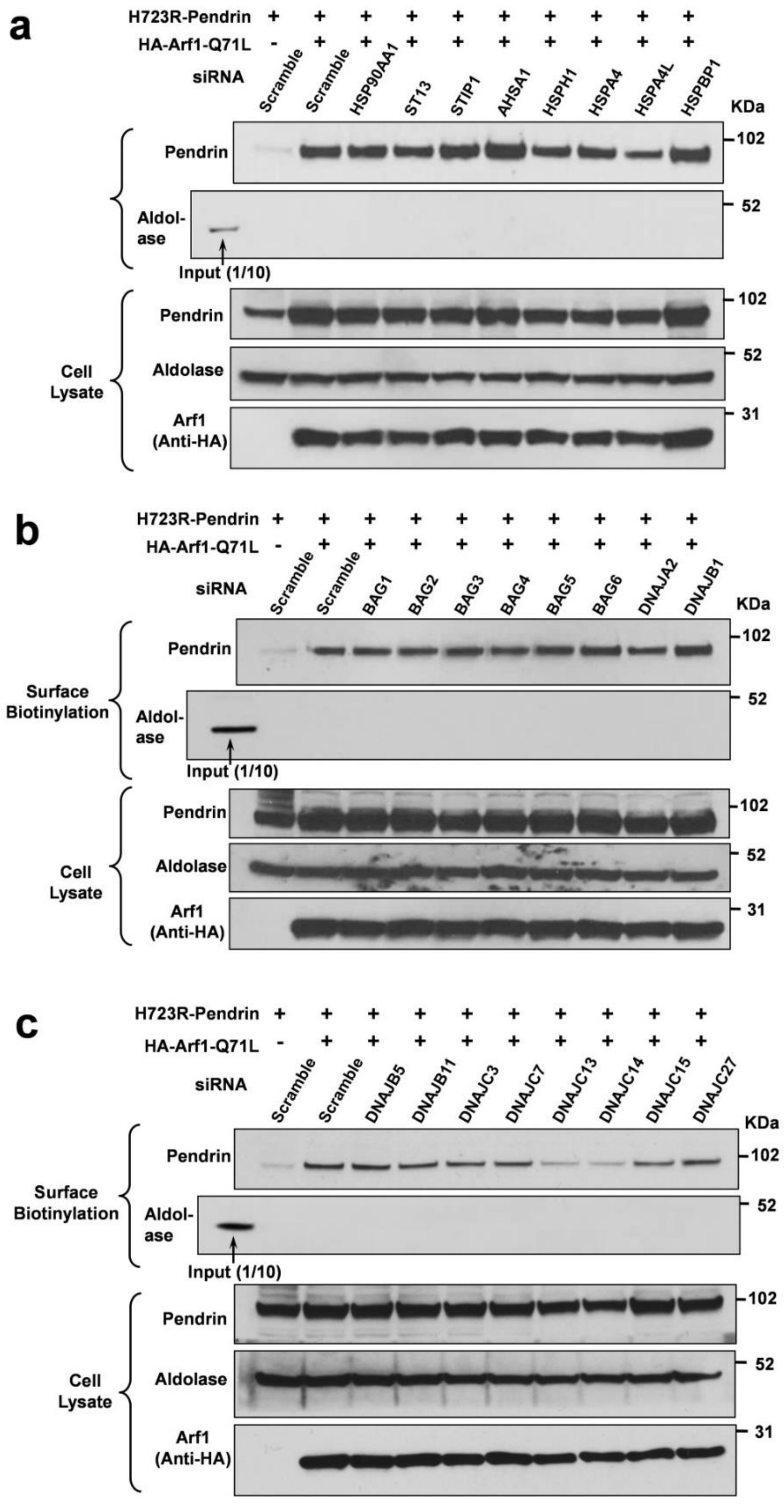

Supplementary Figure 4 | Representative blots of siRNA screen to identify J proteins and nucleotide exchange factors involved in the unconventional transport of H723R-pendrin. Cells were treated with SMARTpool siRNAs against each target gene (50 nM) for 24 h before plasmid transfection. Surface biotinylation assays were performed 24 h after transfection with plasmids encoding H723R-pendrin and Arf-Q71L. A summary of multiple experiments is shown in Figure 5a.

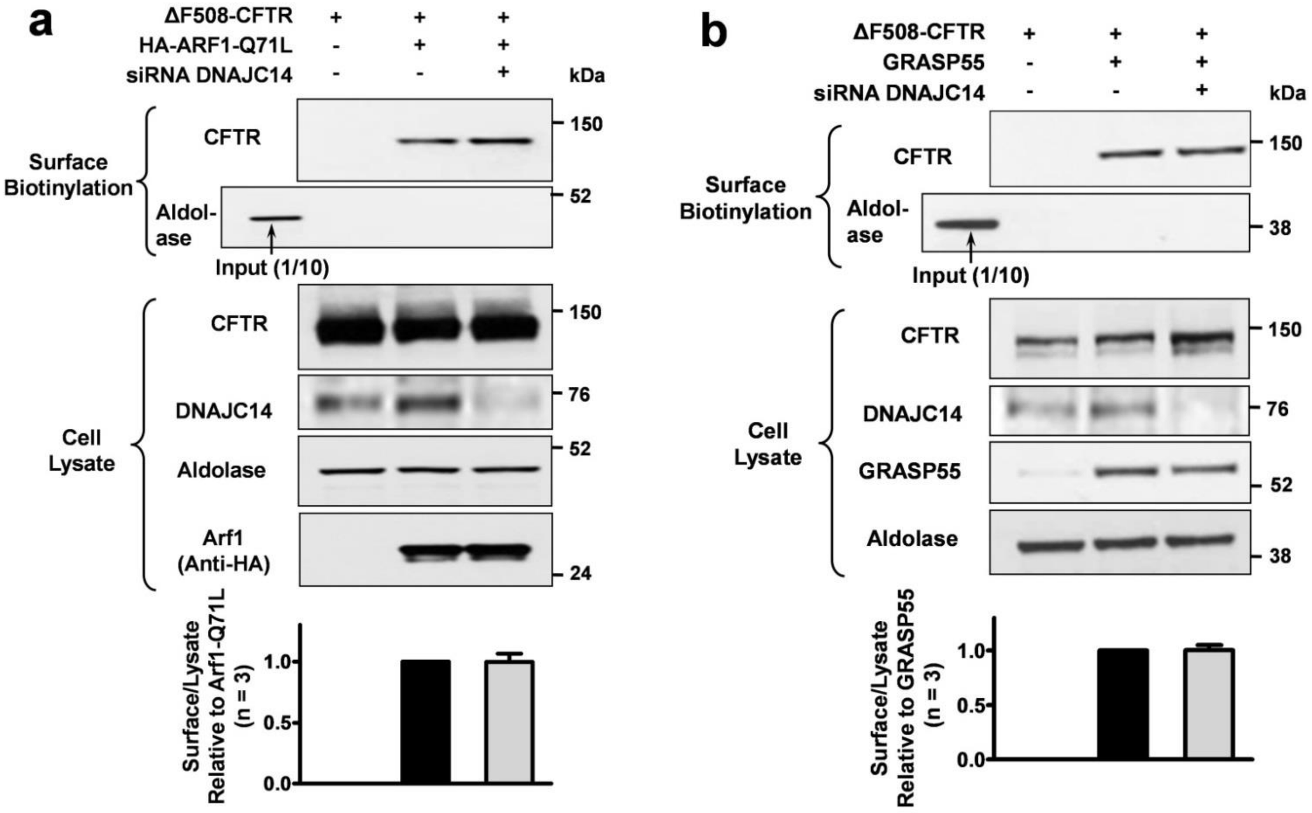

Supplementary Figure 5 | DNAJC14 is not involved in the rescue of ΔF508-CFTR. The rescue of ΔF508-CFTR by Arf1-Q71L **(a)** or GRASP55 **(b)** was not affected by siRNA targeting DNAJC14. Quantitation of multiple experiments is presented under each immunoblot. Other legends are the same as Figure 1. Unprocessed original scans of western blots are shown in Supplementary Fig. 10.

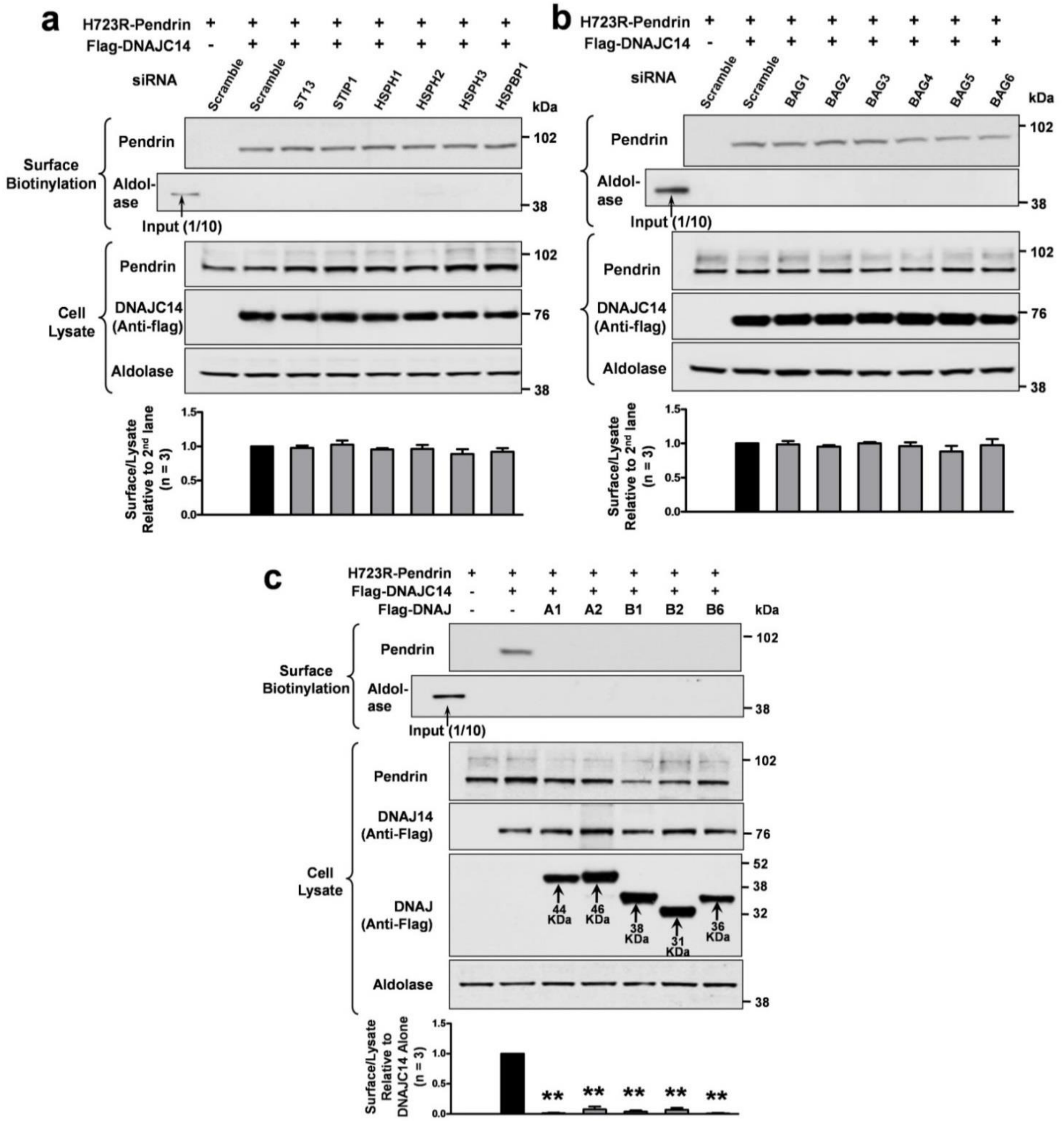

Supplementary Figure 6 | DNAJC14-induced cell-surface expression of H723R-pendrin is not affected by single depletion of co-chaperones and NEFs, but inhibited by coexpression of other J proteins. (**a, b**) PANC-1 cells were treated with siRNAs targeting co-chaperones and NEFs at 24 h before transfection with plasmids expressing H723R-pendrin and DNAJC14. Surface biotinylation assays were performed 24 h after plasmid transfection. Single depletion of co-chaperones and NEFs did not affect DNAJC14-induced cell-surface expression of H723R-pendrin. (**c**) Surface biotinylation assays were performed in PANC-1 cells 24 h after transfection with plasmids expressing DNAJA1, DNAJA2, DNAJB1, DNAJB2, and DNAJB6. Coexpression of other J proteins inhibited the ability of DNAJC14 to rescue cell-surface expression of H723R-pendrin. Quantitation of multiple experiments is presented under each immunoblot. ** $P < 0.01$ by one-way ANOVA, difference from DNAJC14 alone (lane 2), $n = 3$. Unprocessed original scans of western blots are shown in Supplementary Fig. 10.

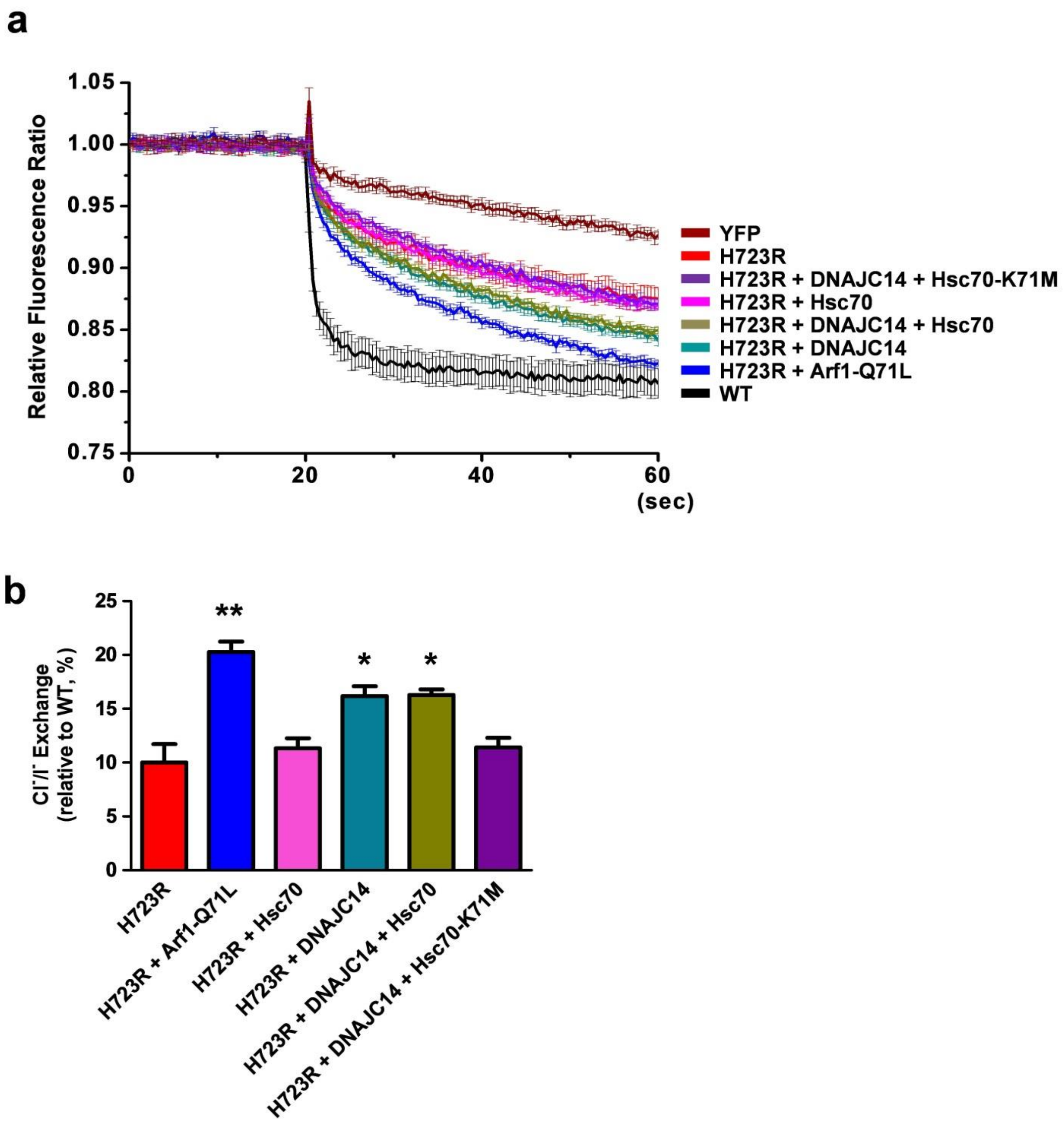

Supplementary Figure 7 | Measurement of Cl^-/I^- anion exchange activity of pendrin. PANC-1 cells were cotransfected with plasmids encoding the indicated proteins and the chloride-sensing yellow fluorescent protein (YFP-H148Q/I152L/F46L). (**a**) Representative traces for Cl^-/I^- exchange measurements are shown. After baseline fluorescence was recorded for 20 sec (400 msec/point), 150 µL of I^- solution (150 mM NaI) was added to each well of a 96-well plate containing 50 µL of Cl^- solution (150 mM NaCl). Fluorescence quenching mediated by intracellular I^- influx was recorded and traced. (**b**) The slopes of fluorescence quenching curves, which represent Cl^-/I^- exchange activity, were calculated by non-linear regression. Signal from cells mock-transfected with plasmid encoding YFP alone was subtracted from each signal and values relative to WT-pendrin were plotted. * $P < 0.05$, ** $P < 0.01$ by one-way ANOVA, difference from H723R alone, $n = 10$.

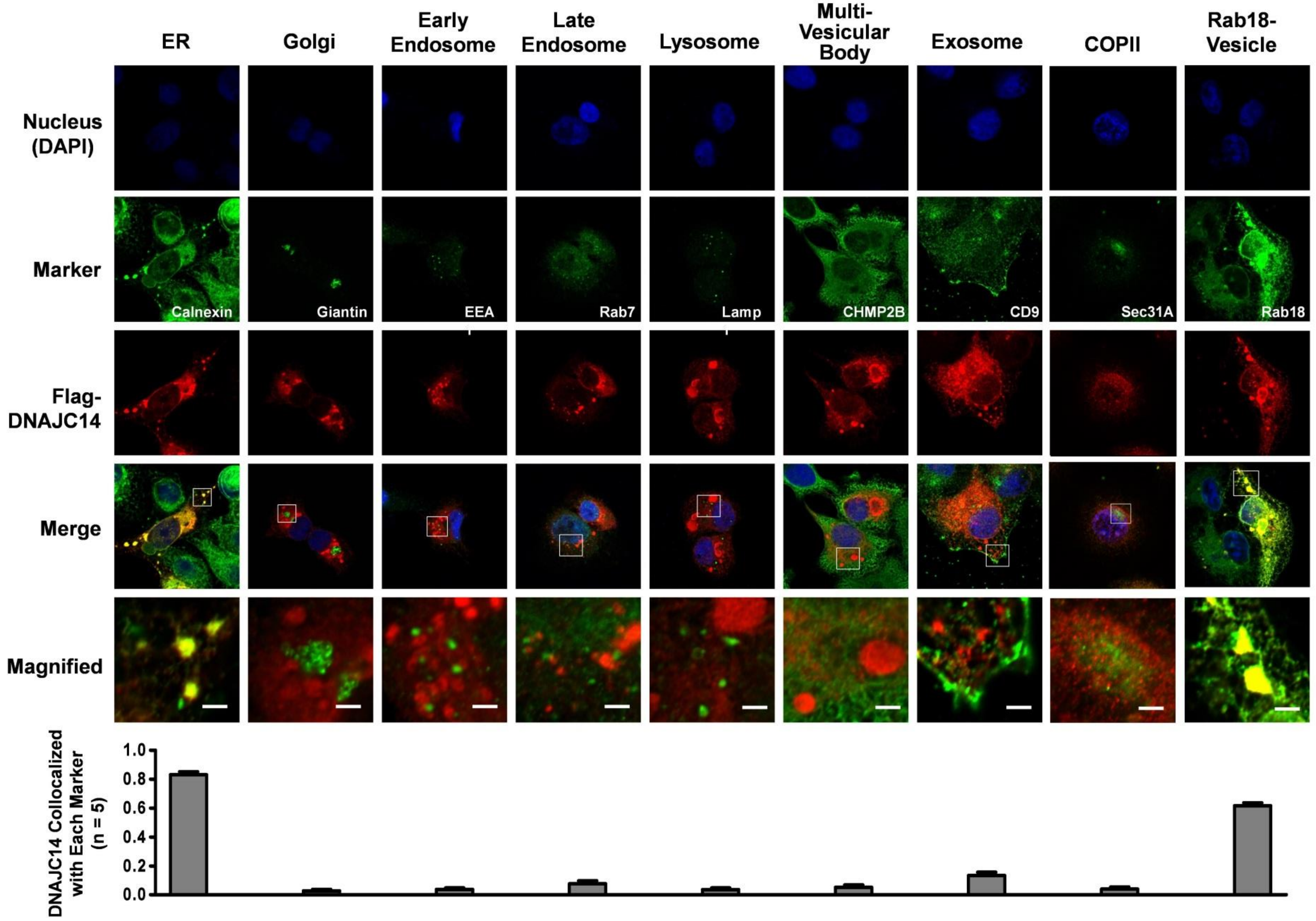

Supplementary Figure 8 | Localization of overexpressed DNAJC14. Cells transfected with Flag-DNAJC14 were co-immunostained with various organelle markers. Representative images are shown in **a**. Quantitation of multiple experiments analyzing the colocalization of DNAJC14 with each marker is shown in **b**. In addition to the ER (calnexin), overexpressed DNAJC14 was localized in some cytosolic punctate regions, where it highly colocalized with Rab18. Regions enclosed by white boxes in the merged images are shown at higher magnification in the bottom row. Nuclei were counterstained with DAPI. Scale bar, 20 μm.

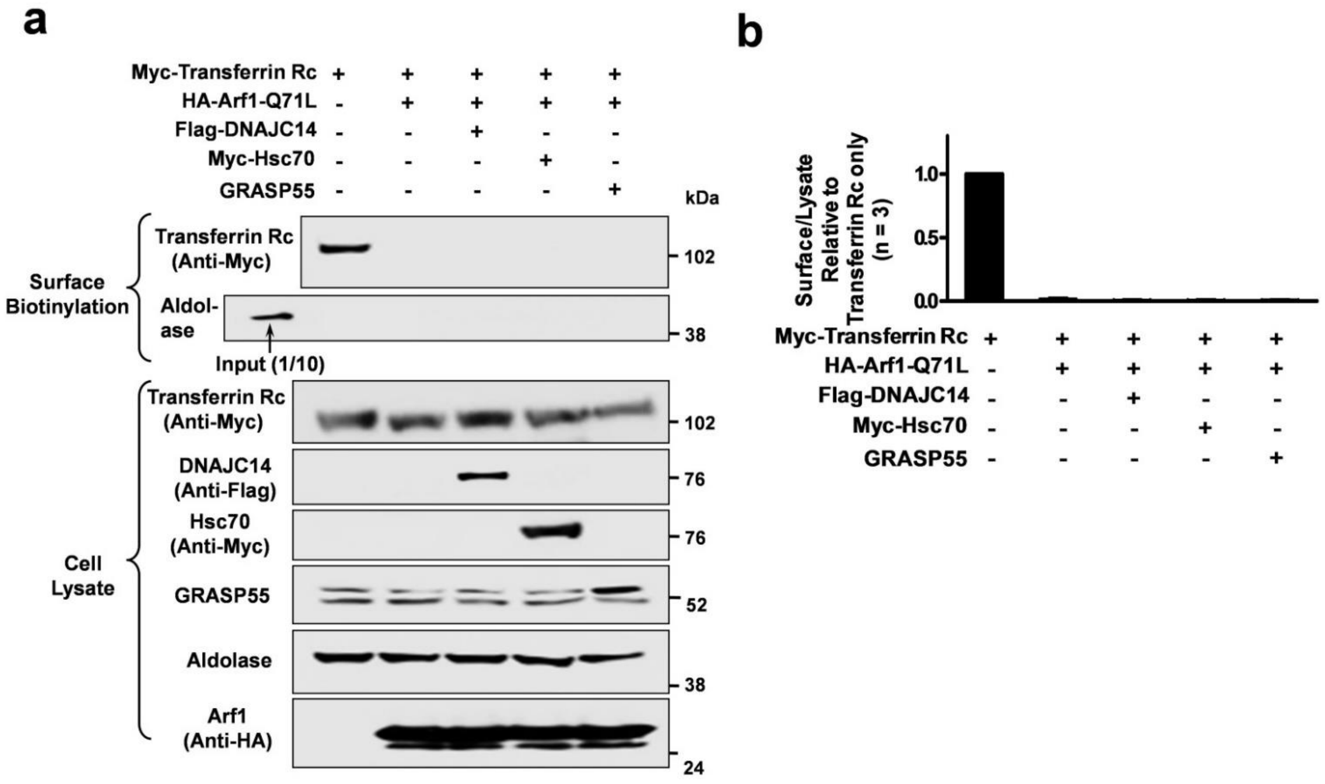

Supplementary Figure 9 | Transferrin receptor does not undergo unconventional secretion. PANC-1 cells were transfected with plasmids expressing transferrin receptor (Rc). Surface biotinylation assays were performed following the co-expression of factors that are known to induce unconventional cell-surface expression of CFTR and pendrin. Representative immunoblot images are shown in **a**. Quantitation of multiple experiments is presented in **b**. Cell-surface expression of transferrin Rc was abolished by the Arf1-Q71L-induced ER-to-Golgi blockade and not rescued by DNAJC14, Hsc70, or GRASP55 overexpression. Unprocessed original scans of western blots are shown in Supplementary Fig. 10.

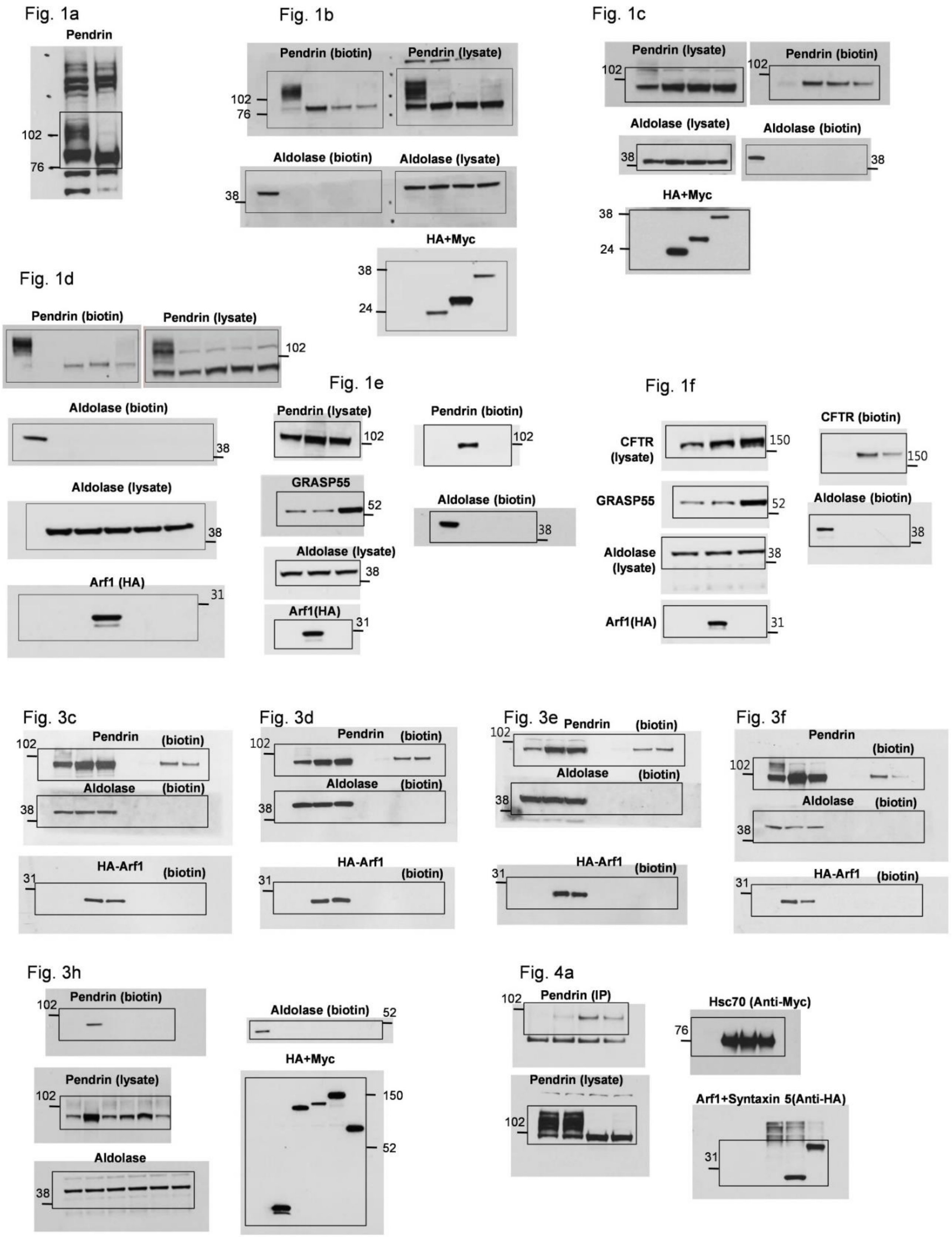

Supplementary Figure 10 | Uncropped images of blots in Figures 1-10 and Supplementary Figures 1-9.

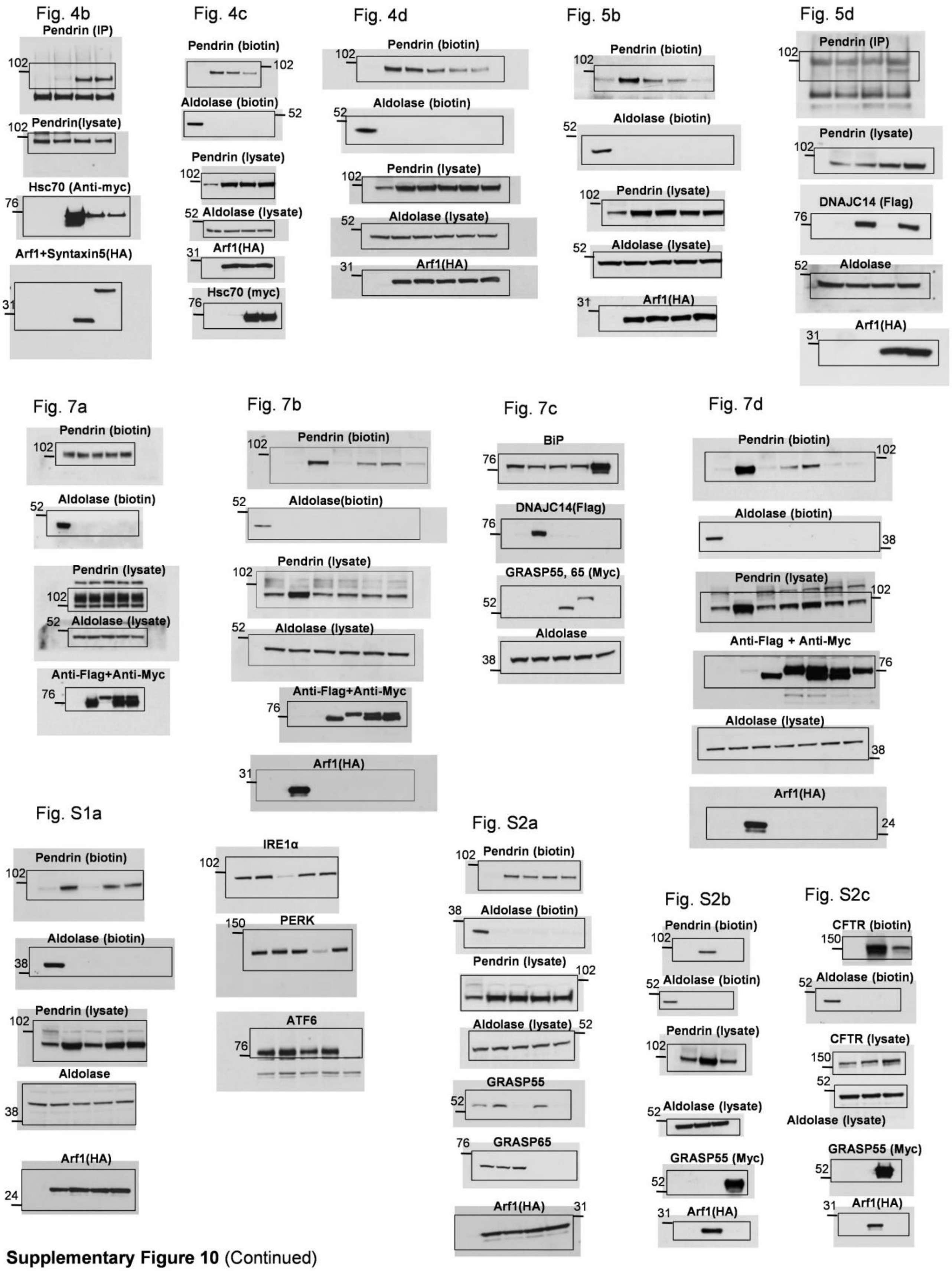

Supplementary Figure 10 (Continued)

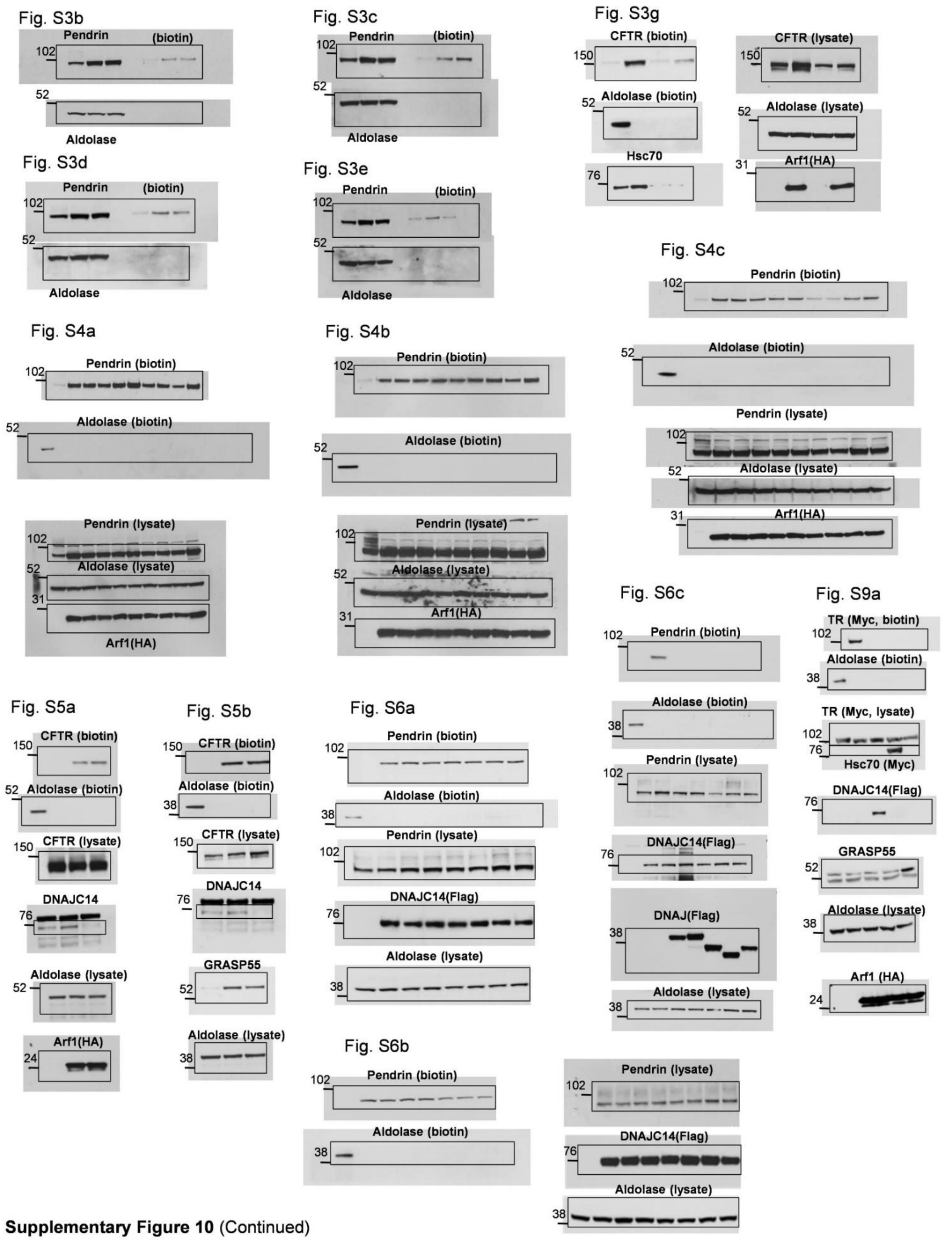

Supplementary Figure 10 (Continued)

Supplementary Table 1 | Binding partners of H723R-pendrin.

Name	GI Number	Amino Acid	Nominal Mass (M_r)	Calculated PI	Mowse Score
**H723R-Pendrin**					
NckAP1	7305303	1128	128707	6.18	75
**H723R-Pendrin + Arf1-Q71L**					
AP3B1	1923268	1093	121146	5.67	42
KIAA0980	119630487	184	21659	6.20	30
Hsc70 (iso1)	5729877	646	70854	5.37	312

Binding partners of H723R-pendrin were analyzed by LC-MS/MS. Among the high Mowse-score proteins, proteins known to be associated with ER quality control or protein trafficking were selected for further investigation. GI number represents sequence identification numbers recorded by the National Center for Biotechnology Information (NCBI) and PI is the abbreviation of isoelectric point. iso1, isoform 1 of Hsc70 (NCBI, NP_006588.1).

Supplementary Table 2 | Oligonucleotide primers used in this study.

**Name**	**Sequence / Catalog Number (Manufacturer)**
**PCR Subcloning**	
Pendrin (WT- and H723R-Pendrin)	
5’ primer	GGG GTA CCA TGG CAG CGC CAG GCG GCA (*Kpn I*, pcDNA3.1)
3’ primer	AAA GCG GCC GCT CAG GAT GCA AGT GTA CGC (*Not I*, pcDNA3.1)
5’ primer	CGG GAT CCA TGG CAG CGC CAG GCG GCA (*BamH I*, TAP)
3’ primer	CCG CTC GAG TCA GGA TGC AAG TGT ACG C (*Xho I*, TAP)
Hsc70	
5’ primer	CCG CTC GAG CAG CAA CCA TGT CCA AGG G (*Xho I*, pCMV-myc)
3’ primer	ATA GTT TAG CGG CCG CTT AAT CAA CCT CTT CAA TGG (*Not I*, pCMV-myc)
NckAP1	
5’ primer	CGG AAT TCA CCA CCA TGT CGC GCT CAG (*EcoR I*, pCMV-myc)
3’primer	GGG GTA CCT TAT GCA GAA GAT GTA AC (*Kpn I*, pCMV-myc)
KIAA0980	
5’ primer	ACG CGT CGA CGA TGG ATG AAG AAG AGA ACC AC (*Sal I*, pCMV-myc)
3’ primer	ATT AGT TTA GCG GCC GCT TAC ACA GAG AGG GCT GCG (*Not I*, pCMV-myc)
DNAJC14	
5’ primer	CCG CTC GAG ACC ATG GCC CAG AAG CAC CCC G (*Xho I*, pCMV-Flag)
3’ primer	GCT CTA GAA CGT TGG AAG GGC CTC CTC AC (*Xba I*, pCMV-Flag)

**Mutagenesis**	
Hsc70-K71M	
5’ primer	TTT GAT GCC A**TG** CGT CTG ATT GGA C
3’ primer	CAA TCA GAC GC**A T**GG CAT CAA AAA CTG TG
DNAJC14-H471Q	
5’ primer	GAT GGT TCA **G**CC TGA CAA AAA T
3’ primer	ATT TTT GTC AGG **C**TG AAC CAT

Supplementary Table 3 | siRNAs used in this study.

Name	Catalog Number (Dharmacon)
NckAP1	L-010640-00
AP3B1	L-017533-00
KIA0980	L-018162-01
Hsc70	L-017609-00
HSP90AA1	L-005186-00
ST13	L-017380-00
STIP1	L-019802-00
AHSA1	L-020014-00
HSPH1	L-004972-00
HSPA4	L-012636-00
HSPA4L	L-017633-00
HSPBP1	L-016607-00
BAG1	L-003871-00
BAG2	L-011961-00
BAG3	L-011957-00
BAG4	L-004379-00
BAG5	L-011960-00
BAG6	L-005062-01
DNAJA2	L-012104-00
DNAJB1	L-012735-01
DNAJB5	L-016492-01
DNAJB11	L-015861-01
DNAJC3	L-012251-00
DNAJC5	L-024098-01
DNAJC7	L-019566-01
DNAJC13	L-010651-01
DNAJC14	L-016792-00
DNAJC15	L-020286-02
DNAJC27	L-009842-00
IRE1a	E-004951-01
PERK	L-004883-00
ATF6	L-009917-00
GRASP55	L-019045-00
GRASP65	L-013510-00

Supplementary Table 4 | Antibodies used in this study.

Name	Catalog number (Manufacturer)	Dilution (Use)
**Primary Antibody**		
anti-pendrin	ab66702 (Abcam)	1:1000 (WB)
anti-calnexin	ab24586 (Abcam)	1:300 (IF)
anti-giantin	ab24586 (Abcam)	1:300 (IF)
anti-Lamp1	ab25630 (Abcam)	1:200 (IF)
anti-CHMP2B	ab33174 (Abcam)	1:200 (IF)
anti-Rab18	ab119900 (Abcam)	1:200 (IF)
anti-GRASP55	ab74079 (Abcam)	1:1000 (WB)
anti-GRASP65	ab30315 (Abcam)	1:1000 (WB)
anti-Hsc70	ab19136 (Abcam)	1:1000 (WB)
anti-ATF6	ab83504 (Abcam)	1:1000 (WB)
anti-DNAJC14	ab121535 (Abcam)	1:100 (IF)
anti-IRE1a	3294P (Cell Signaling)	1:1000 (WB)
anti-PERK	3192S (Cell Signaling)	1:1000 (WB)
anti-Myc	2276S (Cell Signaling)	1:2000 (WB)
anti-HA	2367S (Cell Signaling)	1:2000 (WB)
anti-Rab7	9367 (Cell Signaling)	1:200 (IF)
anti-BiP	3177 (Cell Signaling)	1:1000 (WB)
anti-Sec31A	8506 (Cell Signaling)	1:200 (IF)
anti-pendrin	sc-23779 (Santa Cruz)	1:1000 (WB), 1:200 (IF)
anti-HA	sc-805 (Santa Cruz)	1:1000 (WB)
anti-Myc	sc-789 (Santa Cruz)	1:1000 (WB)
anti-aldolase	sc-12059 (Santa Cruz)	1:1000 (WB)
anti-DNAJC14	sc-368326 (Santa Cruz)	1:500 (WB)
anti-CFTR	05-583 (Millipore)	1:1000 (WB)
anti-Flag	F3165 (Sigma)	1:3000 (WB), 1:300 (IF)
anti-EEA1	610457 (BD biosciences)	1:200 (IF)
anti-CD9	LS-C354601 (LS Bio)	1:200 (IF)
anti-DAPI	D1306 (Thermo)	1:10000 (IF)

Secondary Antibody		
anti-mouse, HRP conjugated	G-21040 (Thermo)	1:2000 (WB)
anti-rabbit, HRP conjugated	32460 (Thermo)	1:2000 (WB)
anti-goat, HRP conjugated	31402 (Thermo)	1:1000 (WB)
Alexa 488, anti-mouse	A11001 (Invitrogen)	1:200 (IF)
Alexa 488, anti-rabbit	A11008 (Invitrogen)	1:200 (IF)
Alexa 488, anti-goat	A11078 (Invitrogen)	1:200 (IF)
Alexa 568, anti-mouse	A11004 (Invitrogen)	1:200 (IF)
Alexa 568, anti-rabbit	A11036 (Invitrogen)	1:200 (IF)
Alexa 568, anti-goat	A11057 (Invitrogen)	1:200 (IF)
Alexa 633, anti-mouse	A21050 (Invitrogen)	1:200 (IF)
Alexa 680, anti-rabbit	A21076 (Invitrogen)	1:200 (IF)
